# Isotope effects observed in diluted D_2_O/H_2_O mixtures identify HOD-induced low-density structures in D_2_O but not H_2_O

**DOI:** 10.1038/s41598-022-23551-9

**Published:** 2022-11-04

**Authors:** Anna Stefaniuk, Sylwester Gawinkowski, Barbara Golec, Aleksander Gorski, Kosma Szutkowski, Jacek Waluk, Jarosław Poznański

**Affiliations:** 1 Institute of Biochemistry and Biophysics Polish Academy of Sciences, Pawińskiego 5a, 02-106 Warsaw, Poland; 2 Institute of Physical Chemistry Polish Academy of Sciences, Kasprzaka 44/52, 01-224 Warsaw, Poland; 3 Adam Mickiewicz University, NanoBioMedical Centre, Wszechnicy Piastowskiej 3, 61-614 Poznan, Poland; 4 Faculty of Mathematics and Science, Cardinal Stefan Wyszyński University, Dewajtis 5, 01-815 Warsaw, Poland

**Keywords:** Chemistry, Physical chemistry, Thermodynamics

## Abstract

Normal and heavy water are solvents most commonly used to study the isotope effect. The isotope effect of a solvent significantly influences the behavior of a single molecule in a solution, especially when there are interactions between the solvent and the solute. The influence of the isotope effect becomes more significant in D_2_O/H_2_O since the hydrogen bond in H_2_O is slightly weaker than its counterpart (deuterium bond) in D_2_O. Herein, we characterize the isotope effect in a mixture of normal and heavy water on the solvation of a HOD molecule. We show that the HOD molecule affects the proximal solvent molecules, and these disturbances are much more significant in heavy water than in normal water. Moreover, in D_2_O, we observe the formation of low-density structures indicative of an ordering of the solvent around the HOD molecule. The qualitative differences between HOD interaction with D_2_O and H_2_O were consistently confirmed with Raman spectroscopy and NMR diffusometry.

## Introduction

Hydrophobicity is a well-known and extensively studied phenomenon^1–4^. It reflects a commonly observed tendency of non-polar molecules to form aggregates, which reduces the contact surface with an aqueous polar environment, thus increasing solvent entropy. Comprehensive studies of hydrophobic interactions at various scales are essential for understanding numerous chemical and biological processes, including protein folding, membrane formation, or water transfer through pores^5–7^. The solvent rearrangement in the proximity of a solute molecule has a vast impact on solvophobic interactions. The hydrophobic effect is manifested mainly by the unfavorable free energy change upon mixing the hydrophobic substances with water^3,8,9^. The introduction of any hydrophobic particle induces unfavorable reorganization of the network of water-water hydrogen bonds. From a thermodynamic point of view, when a non-polar molecule is dissolved in water, there is a considerable loss of entropy and an accompanying increase in enthalpy due to the formation of low-energy and relatively rigid water structures surrounding the solute molecule^10^.

The solute–solvent interactions could be decomposed into two main parts. The first one corresponds to the direct solute–solvent interactions, while the other reflects the solute-induced reorganization of the solvent structure^9,11,12^. However, the solute-induced structure of the hydration shell is expected to be less ordered than that of ice.

The first qualitative description of an aqueous solvent was proposed in 1933. Water is described as a collection of chaotically oriented molecules forming a relatively regular network with oxygen atoms located in nodes. The hydrogen atoms covalently bonded to them are arranged so that they can form a hydrogen bond with a proximal oxygen atom^13^. Then, in 1945, Henry S. Frank and Marjorie Woodard Evans formulated the "iceberg" hypothesis, the first microscopic model of the interaction between water and a dissolved molecule. They proposed that non-polar substances make water form frozen patches or microscopic icebergs around each solute molecule. The water around such a molecule becomes frozen-like, showing properties similar much more to ice than liquid. The extent of the iceberg-like structures increases with the size of the solute molecule. The larger the particles, the larger the icebergs produced in the water, leading to a more significant unfavorable change of dissolving entropy^9,14^.

In 1976, Pierotti presented the scaled particle theory (SPT) based on the statistical analysis of the properties of a solute. The free energy of introducing a hard-sphere solute into a solvent was thus described by scaling up an infinitely small cavity to the desired radius. Therefore, the free energy of solvation is equivalent to the work of forming an empty solute-sized cavity. The SPT combines the microscopic parameters of the solvent with its macroscopic properties, such as temperature dependencies of density or surface tension, so the application of the SPT theory has been limited to relatively small molecules^15–20^. Another theory used in calculating cavity formation in liquids and often compared with the SPT theory is the Sinanoglu theory. Same as SPT theory, it describes the cavity formation process in an aqueous solution which relies heavily on working against the surface tension forces of the solvent. Sinanoglu’s theory is a macroscopic approach based on the thermodynamic properties of pure liquids and diluted solutions. It allows for determining the Gibbs energy and the enthalpy of cavity formation in an aqueous solution. Contrary to the SPT theory, Sinanonglu's theory does not consider an entropic contribution to the cavity formation process^21–23^. Finally, Lum, Chandler, and Weeks proposed the theory of hydrophobic solvation of both small and large non-polar species in water. They assumed that in the case of small molecules, the hydrogen bonding of water is hindered but remains near solutes. In contrast, in the case of large solute molecules, the number of solute–solvent hydrogen bonds is depleted, which leads to the “drying” of extended non-polar surfaces and large forces of solute–solute attraction. According to the LCW model, for small molecules (i.e., with a radius less than 10 Å), the interaction with water is proportional to the volume, while for large molecules to the surface^24–26^.

The structure of water is also studied in silico, and numerous forcefields have been developed^27–30^. Due to the low mass of the proton and the significant role of hydrogen bonds, nuclear quantum effects (NQEs), including tunneling and zero-point energy (ZPE), play an essential role in determining the static and dynamic properties of water^31^. The NQEs can strengthen the hydrogen bond; however, quantum fluctuations facilitate the proton to spread in different directions. Competing nuclear quantum effects are now believed to explain the isotope effects observed in water^31–35^. In general, various force fields are preferably used to simulate particular properties of water systems^36^.

Various stationary and time-resolved vibrational spectroscopy techniques^37–40^ and theoretical studies also extensively studied water structure and internal dynamics^41–43^. Because of the enormous intensity of the OH stretching band, quantitative analysis of the IR absorption of water is challenging^44^. This is not the case with the Raman spectrum. Analysis of Raman spectra is often based on applying multivariate curve resolution (MCR)^38,45–48^, a technique that allows separating the spectrum into contributions from individual components^49,50^. Normal water (H_2_O) and heavy water (D_2_O) are the solvents most commonly used to study the isotope effect. The isotope replacement in a solvent significantly influences the behavior of a single molecule in solution, especially when there are interactions between the solvent and the solute. This effect becomes more significant in D_2_O/H_2_O since the hydrogen bond in H_2_O is weaker than its counterpart, the deuterium bond in D_2_O. Moreover, liquid heavy water is more "structured" than normal water^51,52^.

Using mixtures of normal and heavy water with a significant excess of one isotopologue, e.g., 5% D_2_O in H_2_O or vice versa, allows for observing the OH or OD stretching vibrations decoupled from the stretching vibrations of the environment. For the OH oscillator, studies in dilute solutions of H_2_O in D_2_O lead to HOD molecules being surrounded by D_2_O. Naturally, the opposite occurs when small amounts of D_2_O are added to H_2_O: now, the HOD is solvated by normal water, and the OD oscillator can be regarded as isolated. Applying MCR, it is possible to separate the spectral features of the bulk (pure solvent) from the vibrational modes affected by the presence of the solute. The latter are contained in the so-called solute correlated (SC) spectrum, which includes both the contribution from the isolated oscillator and those solvent molecules perturbed by the solute (HOD). This perturbation extends well beyond the first solvation shell. The number of H_2_O solvent molecules coupled to a single OH oscillator of (solute) HOD has been estimated to be as large as 18^53^.

In our work, we characterize the isotope effect of the solvent, normal water, and heavy water on the solvation of a single HOD molecule. Currently, no experimental data in the literature evidences the partial molar volume of the HOD in D_2_O and H_2_O solutions, respectively. In the presented study, we tested our model of hydrophobic solvation, according to which the solvent density in the solvation shell depends on the solute molecule’s structure and polarity^12,54–56^. Additionally, the excess volume, defined as the difference between the experimentally measured partial molar volume and the in silico calculated molecular volume of a solute, seems to be a reasonable estimator of hydrophobic contribution to protein–ligand binding affinity^57^. We used a simple variant of the MCR technique to assess the disturbance in normal and heavy water structures caused by the HOD molecule. We also determined the diffusion coefficients for proton and deuterium in normal and heavy water.

## Results

### Partial molar volume and the apparent volumetric thermal expansion coefficient of HOD in H_2_O and D_2_O solutions

Our long-term studies have shown that the partial molar volume V_2_^0^, determined from a series of density measurements, could state a measure of the hydrophobic interactions of the dissolved molecule with the aqueous solvent^12^. In general, the larger the difference between V_2_^0^ (the experimental partial molar volume) and the expected molecular volume V_mol_ (calculated in silico from the solute structure), the more significant the hydrophobic effect^54,55^. Recently, we have demonstrated that V_2_^0^ could be used as one of the ADME parameters that describe solute–solvent interactions^57,58^.

**Figure 1:**
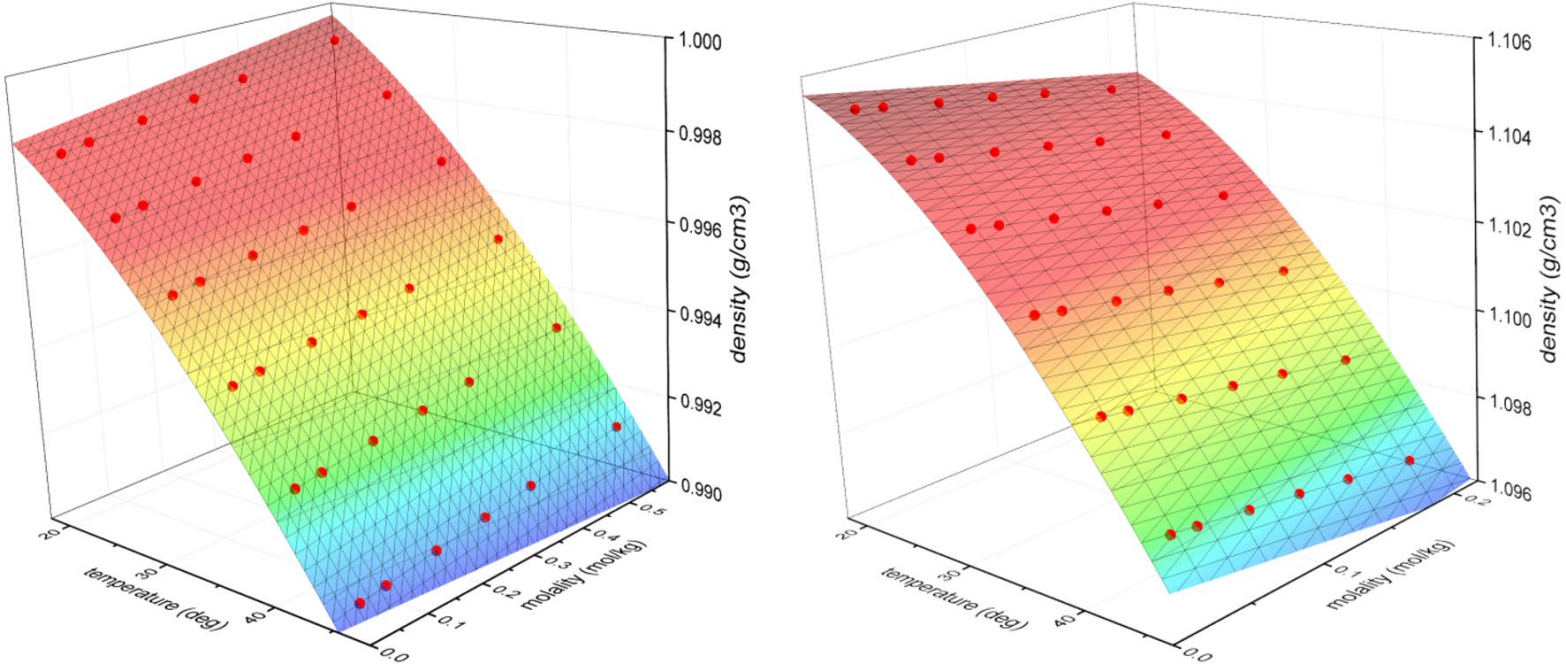
Temperature-dependence of density-molality relationship observed in a single dilution experiment for D_2_O in H_2_O (left) and H_2_O in D_2_O (right). The density dependence on molality measured at six temperatures of 20 to 45 °C was analyzed according to Eqs. (6–9). All data snapshots obtained for four independent series of dilution experiments for D_2_O in H_2_O and H_2_O in D_2_O are shown in Supplementary Figs. S1 and S2, respectively.

The partial molar volume for the HOD molecule in either H_2_O or D_2_O solution was determined from the four dilution series performed at 20–45 °C, analyzed globally according to Eq. (6) (Fig. 1, Supplementary Figs. S1 and S2). The proposed method of data analysis used allowed us to determine the apparent molar volume for both the HOD molecule and the density of pure solvent.

**Figure 2:**
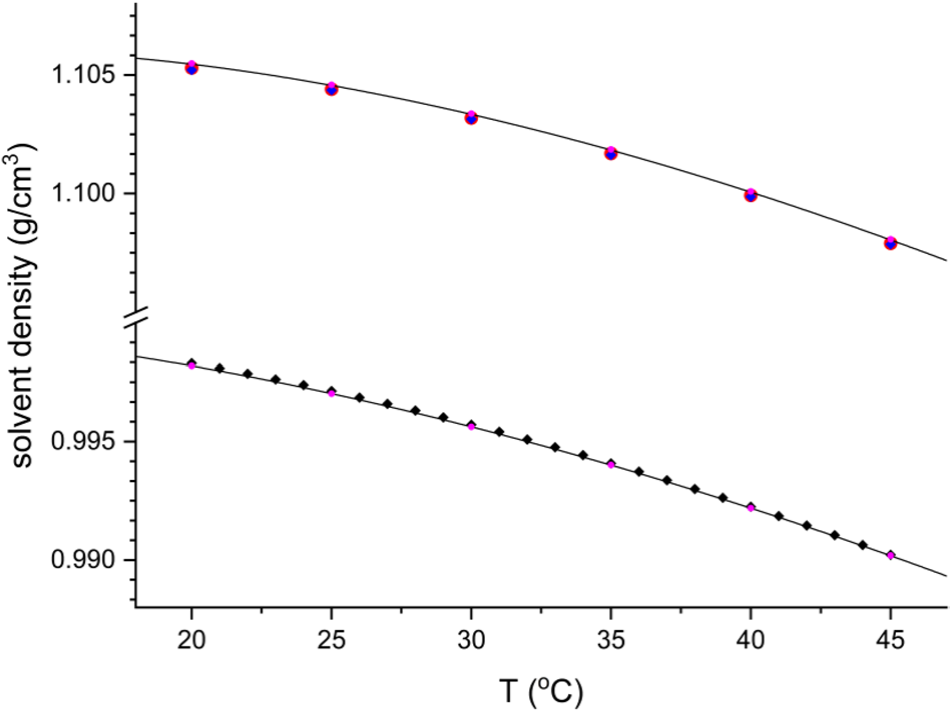
The temperature dependence of the density of pure H_2_O and D_2_O determined in the range of 20–45 °C. The relationships obtained by us (solid lines) are compared with the literature data for H_2_O^59^ (black diamonds) and D_2_O ^60,61^ (black circles). The values estimated from binary H_2_O/D_2_O solvents are denoted in magenta.

The temperature-dependence of the bulk solvent density (either H_2_O or D_2_O) estimated with Eqs. (6–9) from the density data measured for binary H_2_O/D_2_O mixtures is consistent with the literature data (Fig. 2)^59–61^.

The observed agreement between the density measured directly for the pure solvent and the value extrapolated from diluted binary solutions supports the applicability of the proposed method of data analysis. Therefore, the partial molar volume of the solvent molecule in bulk can be estimated directly from the density according to the following equation

1 $${V}_{0}(T)=\frac{{M}_{0}}{{\rho }_{0}(T)}$$

where M_0_ is the molecular mass of the solvent (18.015 and 20.028 g/mol for H_2_O and D_2_O, respectively) and ρ_0_ is the solvent density. The partial molecular volumes at 25 °C are summarized in Table 1. Interestingly, the volume occupied by a single solvent molecule is almost identical for water and heavy water (18.069 and 18.132 cm^3^/mol, respectively), indicating that under normal conditions, the general microscopic organization of heavy water resembles that of the normal “light” water. The volumetric thermal expansion coefficient obtained at 25 °C for both H_2_O (257.30 ± 0.25 × 10^–6^/K) and D_2_O (193.06 ± 0.15 × 10^–6^/K) is consistent with the literature data (α_0_ = 257.21 × 10^–6^ K^-1^)^59^, and (α_0_ = 191.65 × 10^–6^/K)^60^, respectively. It is worth noting that the volumetric expansion coefficient for heavy water is substantially lower than that for “normal” water, thus reflecting differences in the internal dynamics of these two solvents^52,62,63^.

**Table 1:** Experimentally determined thermodynamic parameters: partial molar volume of HOD (V_2_^o^) and solvent (V_0_), thermal volumetric expansivity coefficient of the HOD (α) and bulk solvent (α_0_), and NMR-derived self-diffusion coefficient (D_25_) with the associated activation energy, E_a_. All these values were determined at 25 °C for pure solvents and H_2_O/D_2_O mixtures.

System	V_2_^o^ [cm^3^/mol]	α [10^–6^/K]	V_0_ [cm^3^/mol]	α_0_ [10^–6^/K]	D_25_ [10^−9^m^2^/s]	E_a_ [kJ/mol]
HDO in H_2_O	18.10 ± 0.01	235 ± 15	18.069*	257.11 ± 0.31	2.43 ± 0.02 (^2^H in H_2_O)	18.7 ± 0.4
HDO in D_2_O	20.08 ± 0.05	590 ± 200	18.131*	192.66 ± 0.30	1.81 ± 0.02 (^1^H in D_2_O)	21.4 ± 0.6
H_2_O in H_2_O	–	–	18.069*	257.30 ± 0.25	2.28 ± 0.01^a^ (^1^H in H_2_O)	17.8 ± 0.1^a^
D_2_O in D_2_O	–	–	18.132*	193.06 ± 0.15	2.03 ± 0.01 (^2^H in D_2_O)	18.5 ± 0.3

(*) precision better than 0.001 cm^3^/mol; (a) estimated using data taken from Ref.^64^.

**Figure 3:**
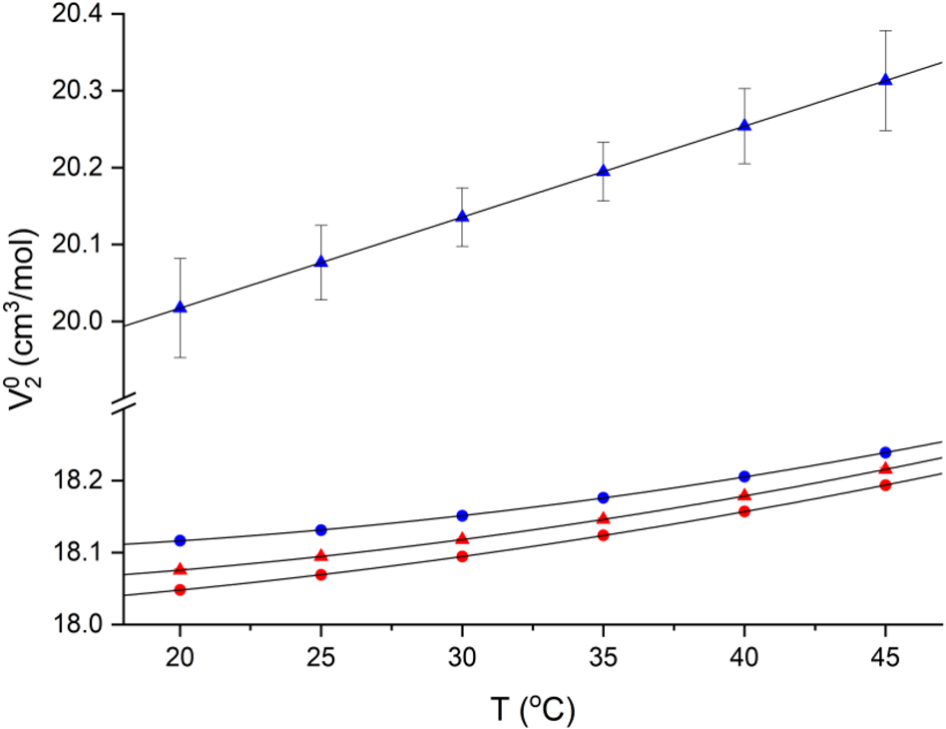
Temperature dependence of the partial molar volume determined from density measurements. The data obtained in H_2_O and D_2_O are in red and blue, respectively. Triangles represent the HOD molecule, while circles denote bulk solvent (H_2_O or D_2_O).

The more spectacular differences are observed for the solute, i.e., the HOD molecule (Fig. 3).

At normal conditions (25 °C), the partial molar volume of HOD in H_2_O resembles that of H_2_O in the bulk solvent (18.10 ± 0.01 vs. 18.07 cm^3^/mol), while in D_2_O is much higher (20.08 ± 0.05 cm^3^/mol). Such a difference should be attributed to the solute-induced reorganization of the proximal solvent molecules rather than to the real solvent-induced change in the solute structure. The observed effect demonstrates that HOD only minutely affects the structure of the surrounding H_2_O molecules, the average organization of which must resemble bulky H_2_O. Contrary, the same HOD molecule strongly affects proximal D_2_O molecules. We have already demonstrated that the apparent expansion of a solute molecule in an aqueous solution, which commonly results from the ordering of water molecules in the solvation shell, is indicative of hydrophobic interactions^57,58^. In this view, the observed excess volume of ~ 2 cm^3^/mol upon virtual transfer of HOD from water to heavy water (i.e., 10% of the apparent HOD volume in H_2_O) indicates relevant “solvophobic” interactions experienced by HOD in D_2_O. Bearing in mind the still proton-deuterium exchange (Eq. 4), one must conclude that the structure of water solvating deuteron resembles that of a bulk solvent. At the same time, the HOD proton substantially affects the structure of the solvating D_2_O molecules.

Furthermore, the apparent volumetric thermal expansivity of the HOD molecule is higher than that of a bulk D_2_O. So, an average structure of proximal D_2_O molecules is more susceptible to temperature-induced changes than a pure solvent (a 2.5-fold increase relative to bulk D_2_O). Interestingly, no such effect is observed in H_2_O (expansivity just between the values for pure H_2_O and D_2_O). So, the local D_2_O structure is much more perturbed by HOD (i.e., proton) than the structure of H_2_O by deuteron.

### Raman spectroscopy

We have applied a simple variant of the MCR technique to assess the perturbations in normal and heavy water structures caused by HDO. For that purpose, Raman spectra have been measured for H_2_O and D_2_O at four different temperatures: 274, 292, 313, and 331 K. The corresponding spectra were registered for mixtures of water and heavy water containing (a) 5% D_2_O in H_2_O; (b) 5% H_2_O in D_2_O. The spectra are shown in Supplementary Figs. S3 and S4. It can be readily observed that the differences between bulk D_2_O and D_2_O containing HOD are much more significant than for the other case (H_2_O compared with H_2_O in the presence of HOD).

**Figure 4:**
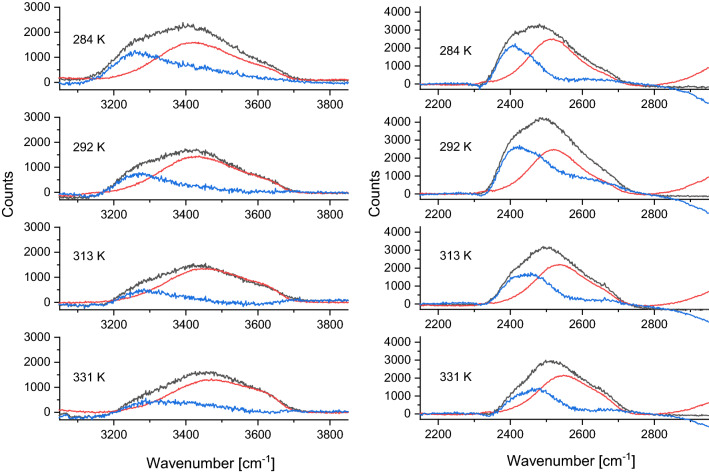
Black, solute-correlated spectra for HOD in D_2_O (left) and H_2_O (right). Red, the contributions from the isolated OD/OH stretching vibration. Blue, the spectrum assigned to solvent molecules perturbed by HOD.

In the next step, the spectra of bulk H_2_O/D_2_O were subtracted from those of the mixtures (Fig. 4.). The resulting solute-correlated spectra consist of (1) contribution from isolated OD or OH and (2) the spectrum of the perturbed solvent shell around HOD. Since the first contribution is known from the spectra of isotopically diluted H_2_O and D_2_O (see Supplementary Figs. S3, S4), it can be subtracted from the corresponding SC curve, yielding the spectrum assigned to HOD perturbed solvent molecules. The comparison of these spectra (blue curves in Fig. 4) unambiguously shows that the intensity of the spectrum originating from the perturbed solvent is much stronger relative to the spectrum of the isolated OD/OH for the case of HDO in D_2_O.

This result suggests that, in agreement with densimetric data, the OD oscillators in D_2_O are more strongly perturbed by HOD than the corresponding OH oscillators in H_2_O. An explanation of this finding can be related to the fact that the OD···O hydrogen bond is considered to be stronger than OH···O^34,65^.

Most IR/Raman papers dealing with water structure focus on the OH stretching region; it has been recently postulated that the bending mode of water can also be used^66^. We found that the analysis of the bending region is more challenging because of the much smaller energy spacing between the bands due to different isotopologues, which results in the spectral overlap.

Since hydrogen bonding may be considered the primary mode of interaction between HOD and the aqueous environment, it is natural to expect that the stronger interaction will lead to a more significant solvent perturbation.

### IR spectroscopy

The attempts to perform a similar analysis for the IR spectra were unsuccessful. It was necessary to obtain very thin (< 2 µm) films of water squeezed in-between two IR windows to obtain reliable IR absorption values (optical density less than 2.0). Using such a procedure, it was impossible to maintain the same optical path length for each sample. Still, the spectra obtained for samples of various solute content (from 2.5 to 10%) could be compared after normalizing to the absorption maximum (Supplementary Fig. S5). We noticed that the differences between pure and isotopically diluted H_2_O spectra were minute, more minor than in the case of Raman spectra. A possible explanation may be related to the fact that the OH stretching band of water contains a contribution from the bending overtone^67,68^. Such contribution is expected to be smaller in the Raman spectrum.

It was also impossible to compare the SC IR spectra in the region of the first overtone of OD and OH. The problem was the overlap, in the OD region, with the combination of the OH stretching and HOH bending modes (from both H_2_O and HOD).

While the more significant perturbation of D_2_O than H_2_O by HOD seems to be well supported by the Raman spectra, the interpretation of temperature dependence requires further, more detailed studies. In agreement with previous reports^69^, the contribution from intermolecular coupling decreases at elevated temperatures. This decrease is monotonous for HDO in H_2_O. However, the situation is more complicated for HDO in D_2_O (Fig. 4). Here, the intermolecular contribution is relatively more significant at 292 K than at 284 K. We checked that this observation is not an experimental artifact caused by laser intensity fluctuation because the intensity of the isolated OH stretch remains constant (within 1%) in the whole temperature range. The origin of this maximum is not clear at present. It may involve modulating hydrogen-bonding strength by thermal activation of torsional and intermolecular stretching modes. To tackle this issue, registration of Raman spectra with small temperature increments is mandatory.

### NMR diffusometry

**Figure 5:**
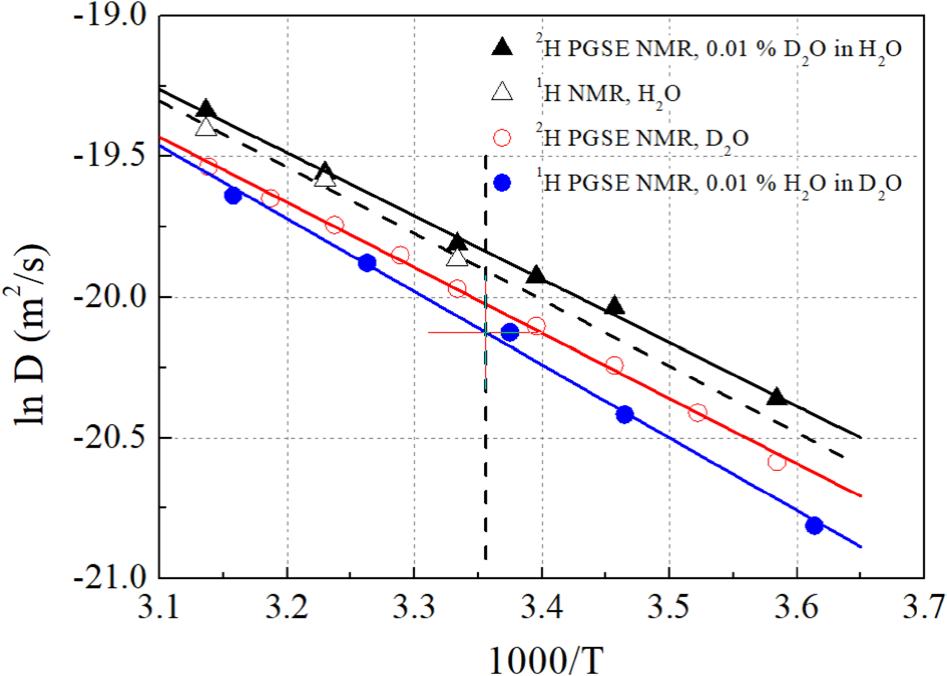
Temperature dependence of self-diffusion coefficients. The activation energies and diffusion coefficients at 25 °C are listed in Table 1. The models fitted to the individual data are shown in Fig. S6.

Self-diffusion coefficients for both proton and deuteron are visibly affected by the isotopic composition of the H_2_O/D_2_O solvent (Fig. 5). Both cations (deuteron and proton) diffuse faster in H_2_O than in D_2_O, and in the same, a solvent deuteron diffuses slightly faster than a proton does. The lowest self-diffusion coefficient is observed for proton in D_2_O. The temperature dependence of self-diffusion enabled the estimation of proton and deuteron behavior in these two environments. The activation energies, E_a_, and the self-diffusion coefficients at 25 °C, D_25,_ are listed in Table 1, The models fitted to the individual data are shown in Fig. S6.

Interestingly, the deuteron/proton self-diffusion coefficients ratio is higher in D_2_O than in H_2_O and increases when the temperature decreases. The latter effect can be directly attributed to the differences in the activation energy for self-diffusion, which is related to the average energy and number of hydrogen bonds formed^70^. Estimated ratio of self-diffusion coefficients at 25 °C (indicated by the vertical dotted line in Fig. 5) for protons in two solvents (1.26 ± 0.02; H_2_O vs. D_2_O) exceeds the value determined in these two solvents for deuteron (1.19 ± 0.01), which indicates that proton is more susceptible to the isotope effect of the aqueous solvent.

## Discussion

The volumetric thermal expansion coefficients experimentally determined at 25 °C for H_2_O and D_2_O are consistent with the literature data^59,60^. What is more, the bulk solvent density (either H_2_O or D_2_O) estimated from the density data measured for binary (H_2_O/D_2_O) mixtures is also consistent with the properties of the pure solvents^59–61^. We identified a qualitative asymmetry between the effect of diluting H_2_O in D_2_O and D_2_O in H_2_O. The density data indisputably show that the HOD molecule induces low-density structures in D_2_O, while no such effect could be observed in H_2_O. Since the partial molar volume of HOD in H_2_O remains the average of the close values determined for bulk H_2_O and D_2_O, the density data indicate that the proton affects the structure of D_2_O to a much greater extent. At the same time, the effect of the deuteron on the H_2_O structure can be assessed as virtually negligible. Therefore, HOD in H_2_O and H_2_O in H_2_O have similar apparent volumes, which differ substantially from the value determined for HOD in D_2_O.

Furthermore, the apparent HOD volume in D_2_O depends on temperature much stronger than in H_2_O. Interestingly, the volumetric thermal expansion coefficient for HOD in H_2_O is close to the average value for bulk H_2_O and D_2_O, indicating the absence of solute-specific perturbations in H_2_O. Contrary to the latter system, the 2.5-fold increase in the volumetric thermal expansion coefficient for HOD in D_2_O implies that the proton-induced low-density structures in D_2_O propagate with the temperature.

Such interpretation agrees with the dynamic picture obtained from NMR diffusometry, in which apparent proton diffusion in D_2_O is slowed down relative to H_2_O much more than is observed for deuteron. Moreover, the inspection of thermal trends demonstrates that the activation energy for HOD in D_2_O exceeds values determined for the three complementary systems, HOD in H_2_O, D_2_O in D_2_O, and H_2_O in H_2_O. Taken together, it could be thus concluded that the HOD diffusion in D_2_O causes a more significant reorganization of the bulk solvent than is required in the three other systems. This effect could be naively assigned to the fact that the amplitude of OH stretching vibrations is minutely larger than that for OD. Hence, the HOD movement in D_2_O (equivalent to H/D location exchange) causes the local pressure increase at a new proton location, rapidly compensated by a local density decrease. Simultaneously, the deuteron in a new location makes a local decrease of pressure, which, however, causes much slower density relaxation due to substantial asymmetry of the Lennard–Jones potential. Consequently, the trajectory of HOD (or proton) diffusing in D_2_O is denoted by “bubbles” of slowly relaxing low-density regions. Such effect is a close analogy to density perturbation near the wingtips, which forms so-called contrail cousins.

The Raman spectroscopy data also confirm that the HOD molecule disturbs the structure of the proximal solvent, and these disturbances are much more significant for heavy water than for normal water. Ben-Amotz has already assigned the 3200 cm^−1^ component of the Raman spectra (blue in Fig. 4) to a low-disorder water characteristic of a highly tetrahedral structure^38^. This supports our interpretation that HOD-induced low-density structures identified in D_2_O display lower disorder than a bulk D_2_O.

## Methods

Samples for all experiments were prepared using heavy water (Merck, UVASOL, deuteration degree min. 99.9% for NMR spectroscopy) and standard (i.e., non-deuterated) water demineralized and filtered and with an ELIX system (Millipore). The samples were always degassed before measurements.

### Density measurements

Partial Molar Volumes (V_2_^0^) of the analyzed system, i.e., H_2_O in D_2_O and D_2_O in H_2_O, were estimated directly based on linear interpolation of the density–molality relationship (Supplementary Figs. S1 and S2) using the high-precision density meter Anton Paar DMA 5000 M equipped with the oscillating u-tube. The density measurements were carried out in the temperature range of 20–45 °C. A molal concentration of the minor component varied between 1 and 10 mM kg^−1^. It never exceeded 0.1%, sufficient to apply a first-order perturbation approach in the data analysis.

The apparent, concentration-dependent molar volume of a solute is defined as:^71^

2 $${V}_{\varphi }=\frac{M}{\rho }+ \frac{{10}^{3}\cdot \left({\rho }_{0}-\rho \right)}{m\cdot \rho \cdot {\rho }_{0}}$$

where m is the molal concentration of a solute (mol/kg), M is the molar mass of solute (g/mol), and ρ and ρ_o_ are the density of the solution and the 'pure solvent', respectively. Consequently, the partial molar volume, ${V}_{2}^{0}$, can be estimated directly from the density data as the volume of a solute at the infinite dilution, according to Eq. (3)^57^

3 $${V}_{2}^{0}={V}_{\varphi }^{m\to 0}= \frac{M}{{\rho }_{0}}-\frac{{10}^{3}}{{\rho }_{0}^{2}}\cdot {\left.\frac{\partial \rho }{\partial m}\right|}_{m\to 0}$$

where ρ_o_ and ∂ρ/∂m are the intercept and slope for the linear approximation of the ρ(m) relationship, respectively.

In the mixture of H_2_O and D_2_O, hydrogen and deuterium atoms (^1^H, ^2^H) are rapidly exchanged between the solvent and solute molecules and, consequently, the following reaction

4 $${\text{H}}_{{2}} {\text{O}} + {\text{D}}_{{2}} {\text{O}} \rightleftarrows {\text{2HDO}}$$

takes place, leading to the formation of HOD molecules (semiheavy water)^72^. So, Eqs. (2) and (3) had to be modified upon the exchange process, considering the mass balance associated with Eq. (4). Therefore, the H_2_O/D_2_O mixture consists of three components: H_2_O, D_2_O, and HDO. However, in highly diluted H_2_O/D_2_O or D_2_O/H_2_O mixtures, the minor isotopic form (either H_2_O or D_2_O) remains negligible. In such conditions, the apparent molar volume attributed to the semiheavy water, ${V}_{\varphi }^{HOD}$, equals:

5 $${V}_{\varphi }^{HOD}=\frac{{M}_{0}}{{2\cdot \rho }_{0}}+\frac{M}{2\cdot \rho }+\frac{{10}^{3}}{2\cdot \rho {\cdot \rho }_{0}}\cdot \frac{{\rho }_{0}-\rho }{m}$$

where m is the molal concentration of the solute (mol/kg), M_o_ is the molar mass of the solvent, M is the molar mass of the solute, and ρ and ρ_o_ are the density of the solution and the bulk solvent, respectively.

Consequently, the partial molar volume of the HOD molecule, ${V}_{2}^{0}(\text{HOD})$, can be estimated as:

6 $${V}_{2}^{0}(HOD)=\frac{{M}_{0}+M}{{2\cdot \rho }_{0}}-\frac{{10}^{3}}{2\cdot {\rho }_{0}^{2}}\cdot {\left.\frac{\partial \rho }{\partial m}\right|}_{m\to 0}$$

where ρ_o_ and ∂ρ/∂m state the intercept and slope for the linear approximation of the ρ(m) relationship.

The density measurements were carried out at the temperature range of 20–45 °C. Such an approach allowed the determination of the apparent thermal volumetric expansivity of the HOD molecule. Independently, the thermal volumetric expansivity of the bulk solvent (either H_2_O or D_2_O) can be estimated from the density data extrapolated to infinite solute concentration at different temperatures. Because of a limited temperature range sampled, we have used a first-order approximation for the V_2_^0^(T) relationship, assuming the temperature-independent volumetric thermal expansion coefficient α_HOD_ (Eq. 7).

7 $${{\upalpha }}_{\text{HOD}} =\frac{1}{{V}_{2}^{0}} \cdot \frac{\partial {V}_{2}^{0}}{\partial T}$$

The algorithm based on Eq. (6) was implemented in Origin (version 9.9; www.originlab.com). The model parameters were fitted globally to all dilution series (either D_2_O in H_2_O or H_2_O in D_2_O), assuming for HOD molecules the global values of V_2_^0^(T_ref_) at T_ref_ = 25 °C and the associated thermal volumetric expansivity coefficient, α_HOD_.

8 $${V}_{2}^{0}(T) = {V}_{2}^{0}({T}_{ref}) \cdot (1 + {\alpha }_{HOD}\cdot (T-{T}_{ref}))$$

Finally, the resulting changes in the bulk solvent density, ρ_0_(T), were analyzed according to the third-order polynomial.

9 $${\uprho }_{0}\left(\text{T}\right)= {p}_{0}+{p}_{1}\cdot (T-{T}_{ref})+{p}_{2}\cdot {(T-{T}_{ref})}^{2}+{p}_{3}{(T-{T}_{ref})}^{3}$$

The thermal volumetric expansivity of a bulk solvent, α_0_(T), was further estimated directly from the temperature-induced variation of the pure solvent density ρ_0_(T).

10 $${{\upalpha }}_{0}(\text{T}) = \frac{-1}{{\rho }_{0}}\cdot \frac{\partial {\rho }_{0}}{\partial T}= \frac{-{p}_{1}-2{p}_{2}\cdot (T-{T}_{ref})-3{p}_{3}\cdot {(T-{T}_{ref})}^{2}}{{p}_{0}+{p}_{1}\cdot (T-{T}_{ref})+{p}_{2}\cdot {(T-{T}_{ref})}^{2}+{p}_{3}\cdot {(T-{T}_{ref})}^{3}}$$

We have additionally tested that the apparent coefficient of thermal volumetric compressibility coefficient, $\frac{1}{{V}_{\varphi }^{HOD}}\cdot \frac{\partial {V}_{\varphi }^{HOD}}{\partial T}$, does not depend on the solute concentration. This observation justifies the global analysis neglecting the second-order correction for solute–solute interactions.

### Raman Spectra

Raman measurements were made for pure H_2_O, pure D_2_O, and mixtures of 5% D_2_O in H_2_O and 5% H_2_O in D_2_O. Raman scattering spectra were measured on the InVia Renishaw microspectrometer equipped with a macroscopic adapter. Measurements were made using the 632.8 nm line of a HeNe laser (linear polarization 100:1), a 1200 l/mm grating, and a CCD camera as a detector. The "continuous scan" mode was used, in which the grating rotates synchronously with the shift of charges on the pixels of the CCD camera. Spectra were recorded in the 800–4000 cm^−1^ range. Accumulation for a single measurement was set to 10 s, and the laser power on the sample was 2 mW. Water mixtures were measured in a 10 mm cuvette using a 30 mm lens in a back-scattering configuration. The cuvette was thermostated to an accuracy of 0.1 K using a NESLAB RTE200 flow thermostat. The measurements were made at 284, 292, 313, and 331 K. Every sample was stabilized for about one hour after any temperature change. Ten spectra were recorded at each temperature for each mixture, then the artifacts due to cosmic rays were manually removed, and all ten spectra were averaged.

### Infrared spectroscopy (IR spectra)

IR measurements were carried out for pure H_2_O and D_2_O, and 2.5%, 5%, 7.5%, and 10% mixtures of D_2_O in H_2_O and, vice versa, H_2_O in D_2_O. IR spectra (1 cm^−1^ spectral resolution) were recorded on a Nicolet Magna-560 FTIR instrument equipped with an MCT/B liquid N_2_-cooled detector. In order to obtain a thin layer of the investigated mixture, a small drop of solution was placed between two CaF_2_ windows. Afterward, two windows without a spacer were tightly pressed against each other in a home-built IR cell. The optical path (a thickness of water film between two windows) was calculated based on the molar absorption coefficient of water^73^. The obtained thickness of the water film varied for different experiments from 1.4 to 2 µm.

### NMR diffusometry

The self-diffusion coefficients of D_2_O in H_2_O were measured using ^2^H PGSE Dbppste pulse sequence on Agilent DD2 600 MHz spectrometer (Santa Clara, California, USA) equipped with DOTY DSI-1372 multinuclear probe-head with a maximum magnetic field gradient of 30 T/m (Doty Scientific, Clemson Rd, Columbia, USA). The self-diffusion coefficients of H_2_O in D_2_O were measured using ^1^H PGSE Dbppste^74^ using Agilent DD2 800 MHz spectrometer. The exact temperatures were calibrated using ethylene glycol by analyzing the chemical shift difference between CH_2_ (ethylene glycol) and OH groups according to the Bruker VT-Calibration Manual. Gradient calibration constants on both spectrometers were determined using a water sample obtained from Mili-Q using the Agilent gradient calibration procedure at 21 °C.

NMR samples were poured into 5 mm NMR sample tubes (0.01% D_2_O in H_2_O and 0.01% H_2_O in D_2_O). The spin-echo intensities were fitted according to the Stejskal-Tanner equation^75^. using MestReNova 14 software (https://mestrelab.com/software/mnova/).

The self-diffusion coefficient at T_0_ = 25 °C, D_25_, and the activation energy, E_a_, were estimated according to the modified Arrhenius Eq. (11) implemented in Origin (version 9.9; www.originlab.com).

11 $$\text{D}\left(\text{T}\right)={\text{ D}}_{25}\cdot \text{exp}\left(\frac{{\text{E}}_{\text{a}}}{\text{R}}\cdot \frac{T-{T}_{0}}{\text{T}\cdot {T}_{0}}\right)$$

## Supplementary Information

Supplementary Figures.

## Data Availability

The raw density data, Raman spectra, and NMR data collected and analyzed during the current study are available from the corresponding author upon reasonable request.
