# Supplementary material for: Isotope effects observed in diluted D2O/H2O mixtures identify HOD-induced low-density structures in D2O but not H2O
Source: Sci Rep. 2022 Nov 4;12:18732. doi: 10.1038/s41598-022-23551-9 (PMC9636167; doi:10.1038/s41598-022-23551-9)
Supplement: Supplementary file 1 — Supplementary Figures. [file 41598_2022_23551_MOESM1_ESM.docx]

**Supplementary material for:**

**Isotope effects observed in diluted D_2_O/H_2_O mixtures identify HOD-induced low density structures in D_2_O but not H_2_O.**

**Anna Stefaniuk**^1^**, Sylwester Gawinkowski**^2^**, Barbara Golec**^2^**, Aleksander Gorski**^2^**, Kosma Szutkowski**^3^**, Jacek Waluk**^2,4^**, and Jarosław Poznański**^1,*^

^1^ Institute of Biochemistry and Biophysics Polish Academy of Sciences, Pawińskiego 5a, 02-106

Warsaw, Poland;

^2^ Institute of Physical Chemistry Polish Academy of Sciences, Kasprzaka 44/52, 01-224 Warsaw,

Poland;

^3^ Adam Mickiewicz University, NanoBioMedical Centre, Wszechnicy Piastowskiej 3, 61-614 Poznan,

Poland;

^4^ Faculty of Mathematics and Science, Cardinal Stefan Wyszyński University, Dewajtis 5, 01-815

Warsaw, Poland

^*^Correspondence to be addressed to: [jarek@ibb.waw.pl](mailto:jarek@ibb.waw.pl)


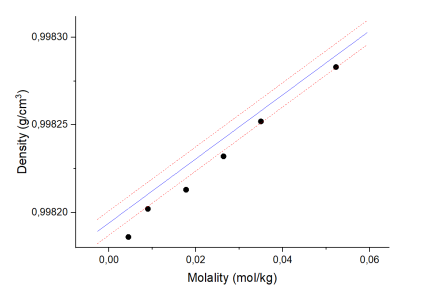

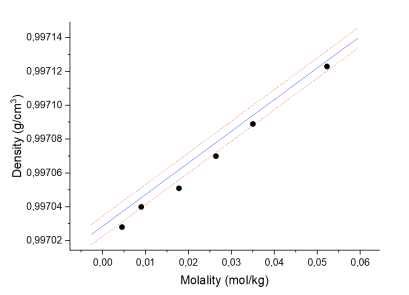

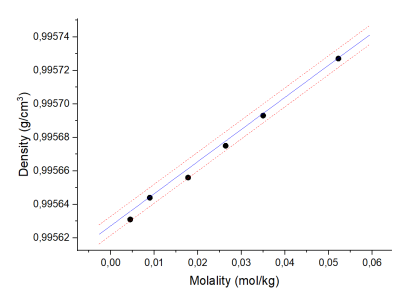


**
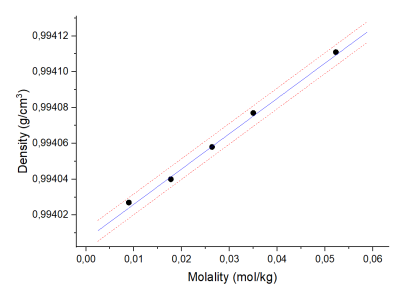

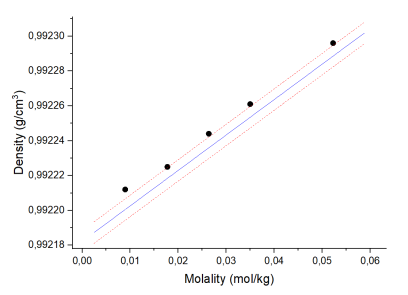

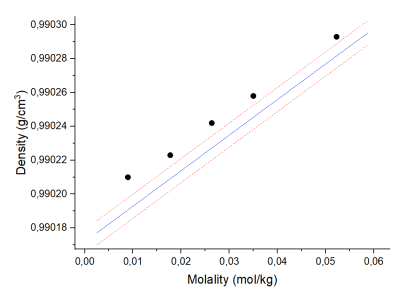
**

**
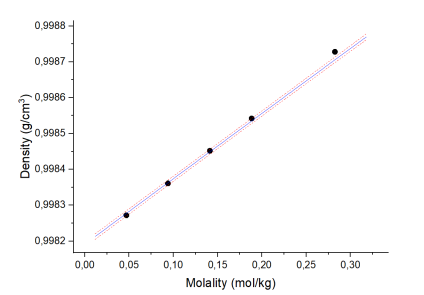

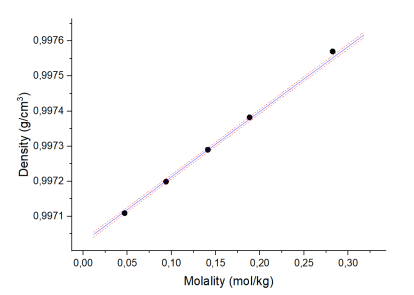

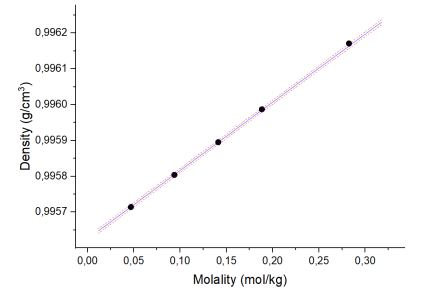
**

**
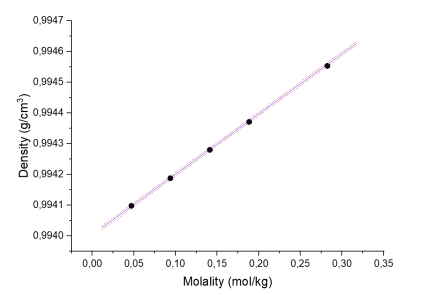

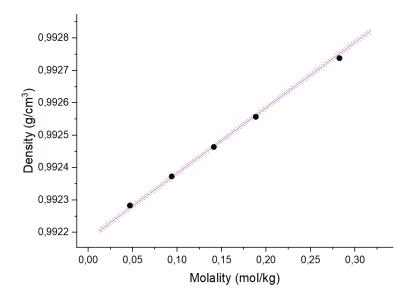

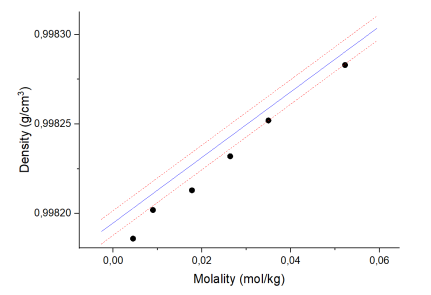
**

**
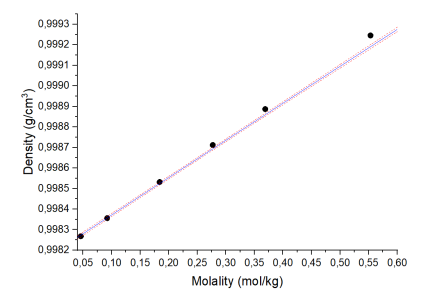

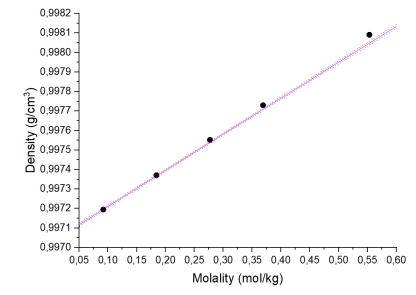

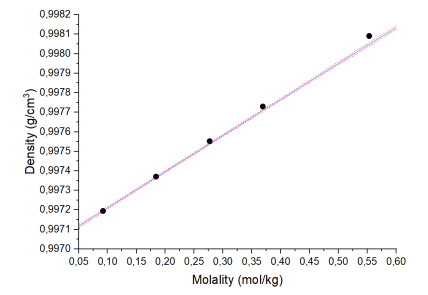
**

**
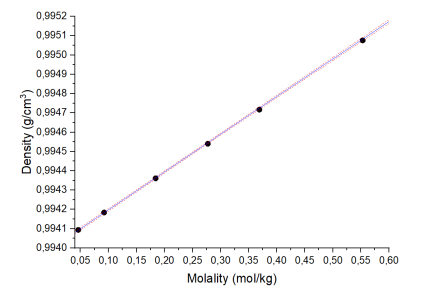

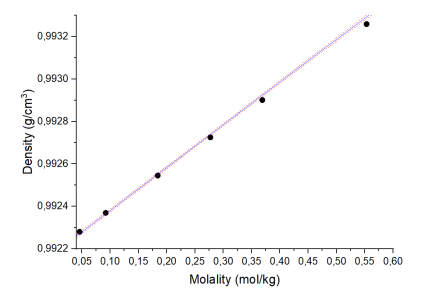

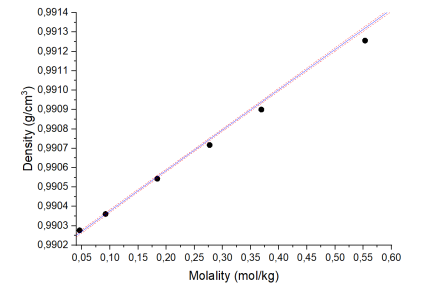
**

**
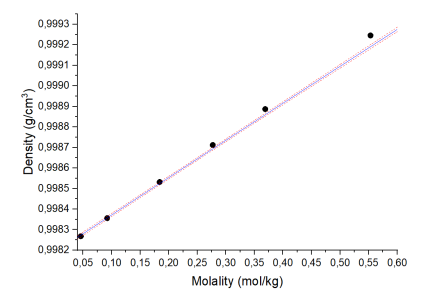

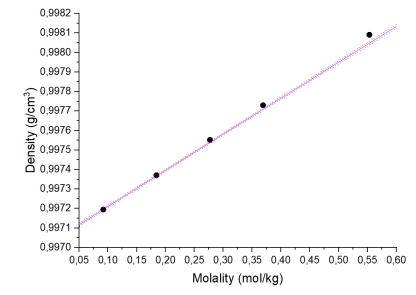

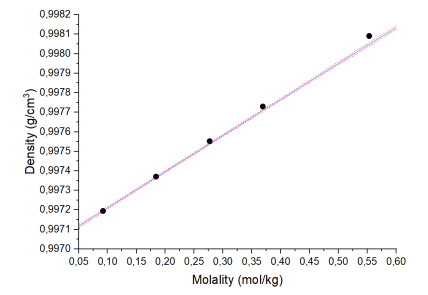
**

**
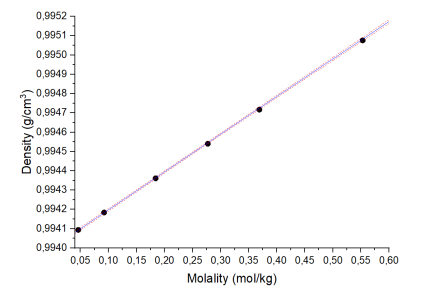

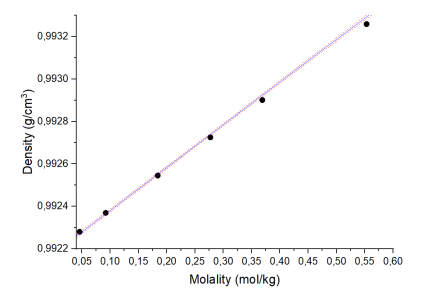

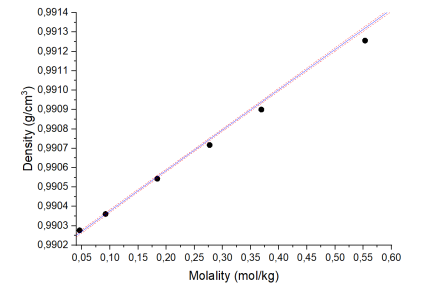
**

**Figure S1.** Density - molality relationship for HDO in H_2_O measured experimentally for 6 temperatures in range of 20 to 45^o^C. The results of four independent experiments are shown.


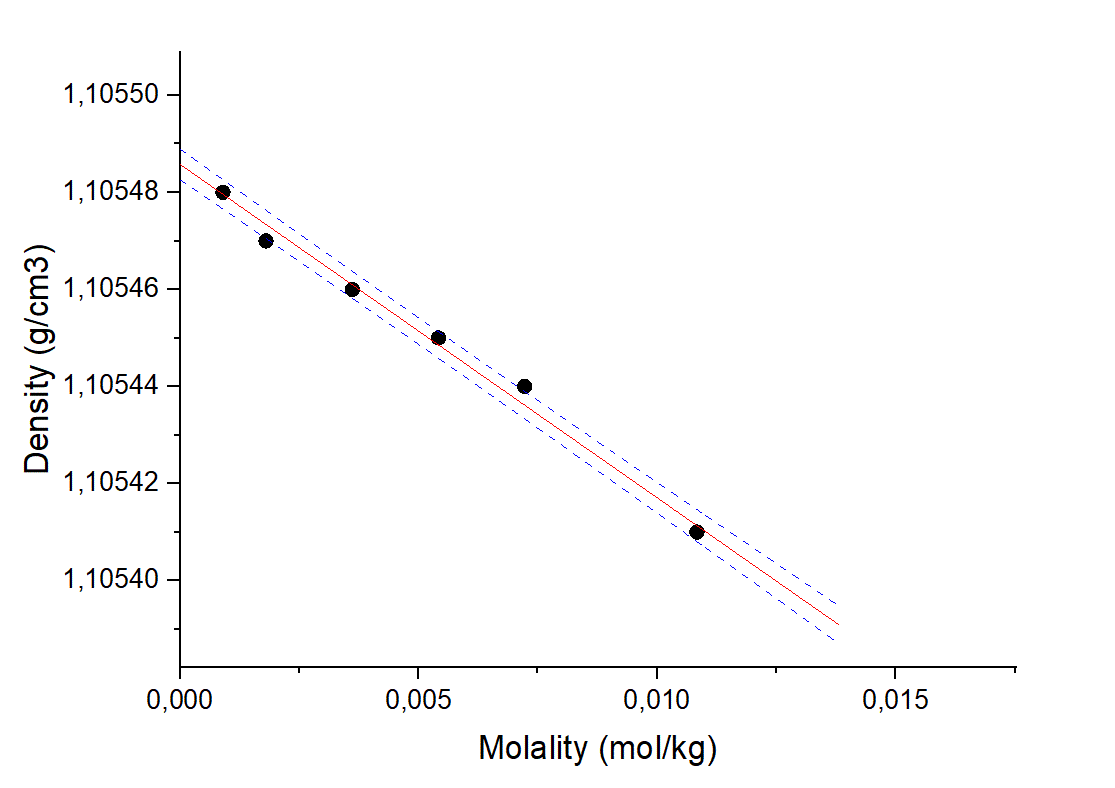

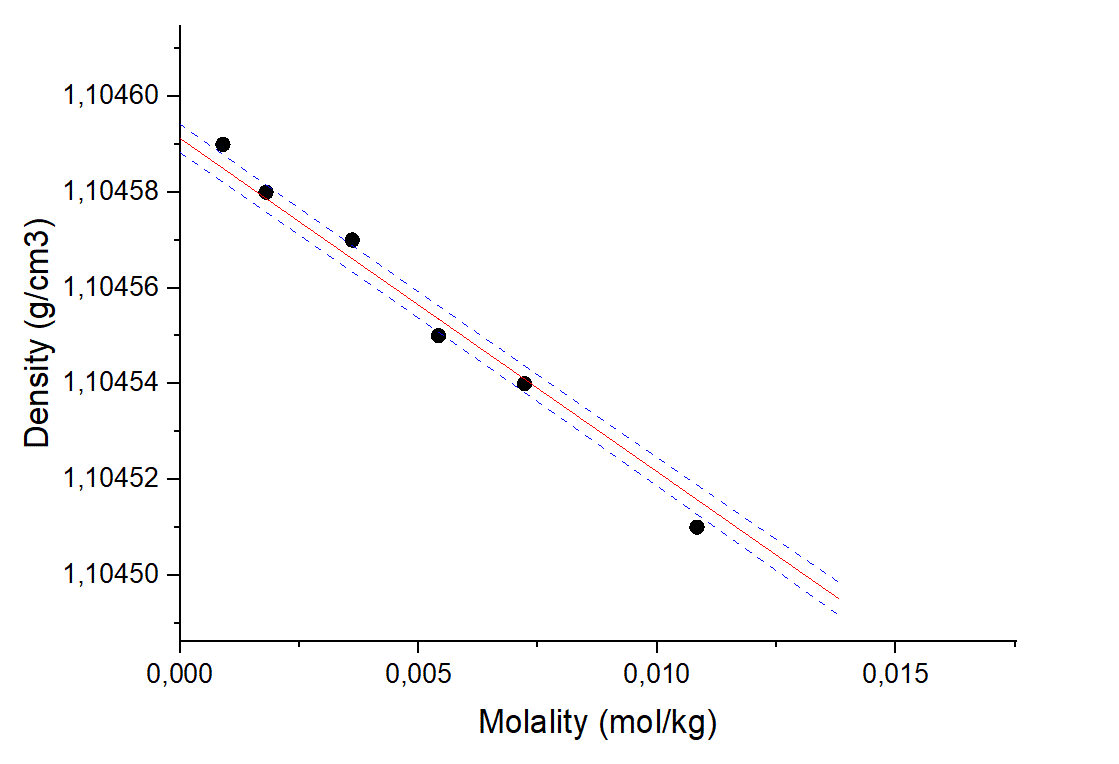

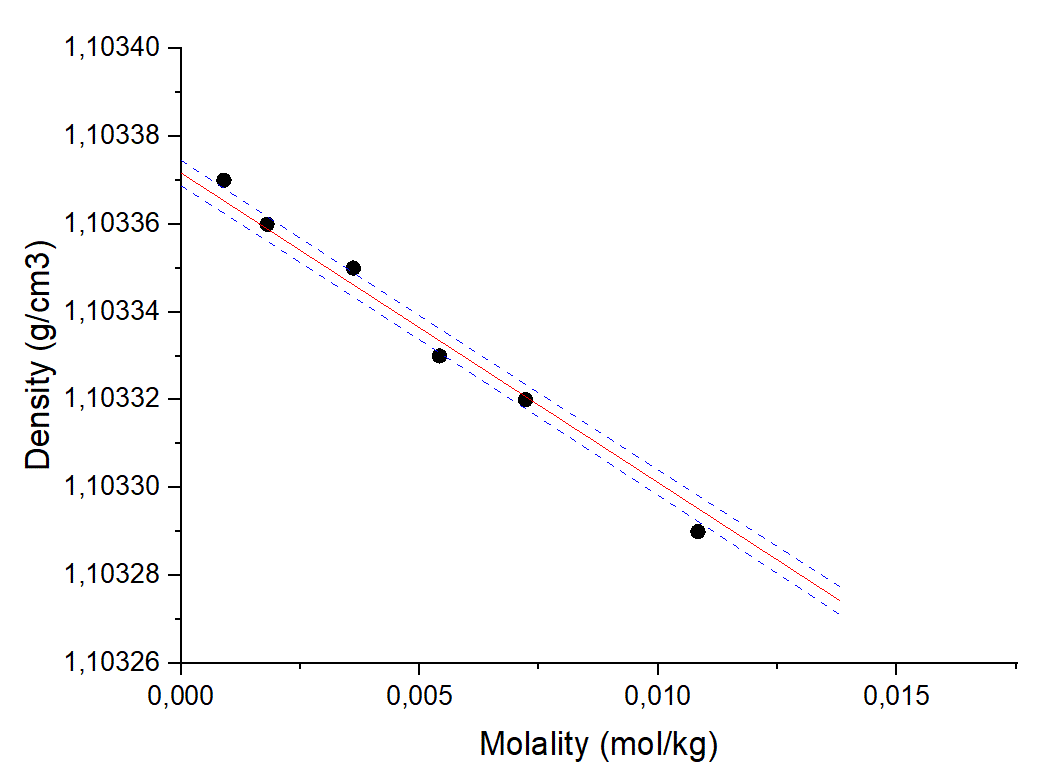


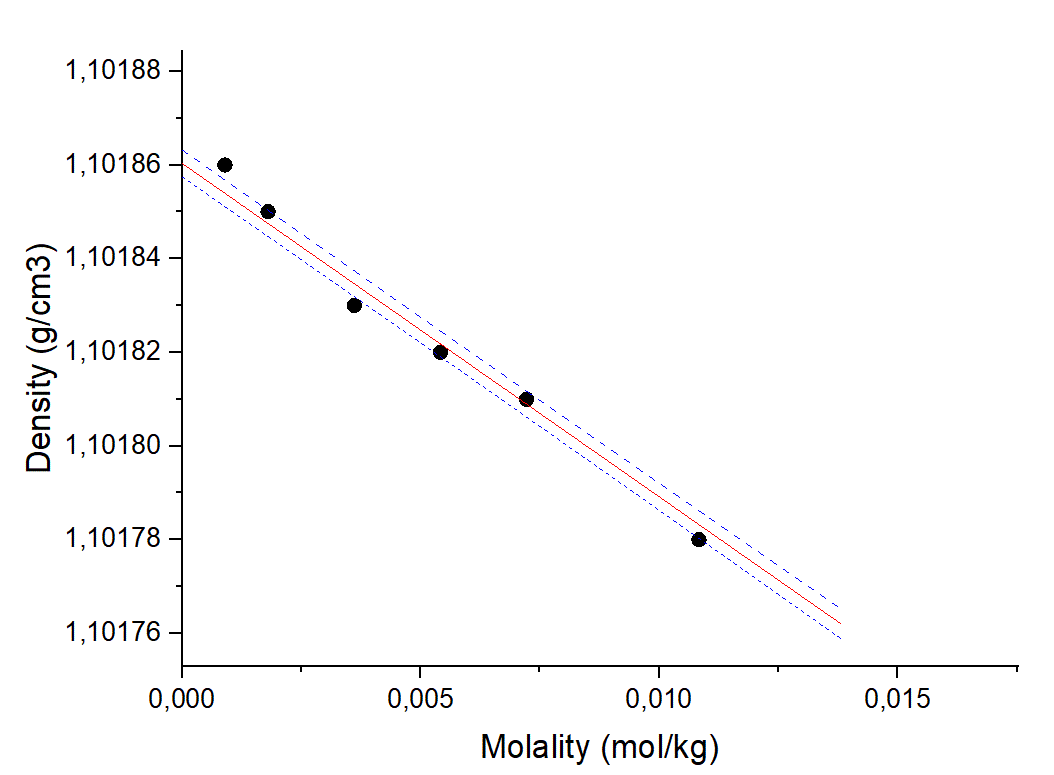

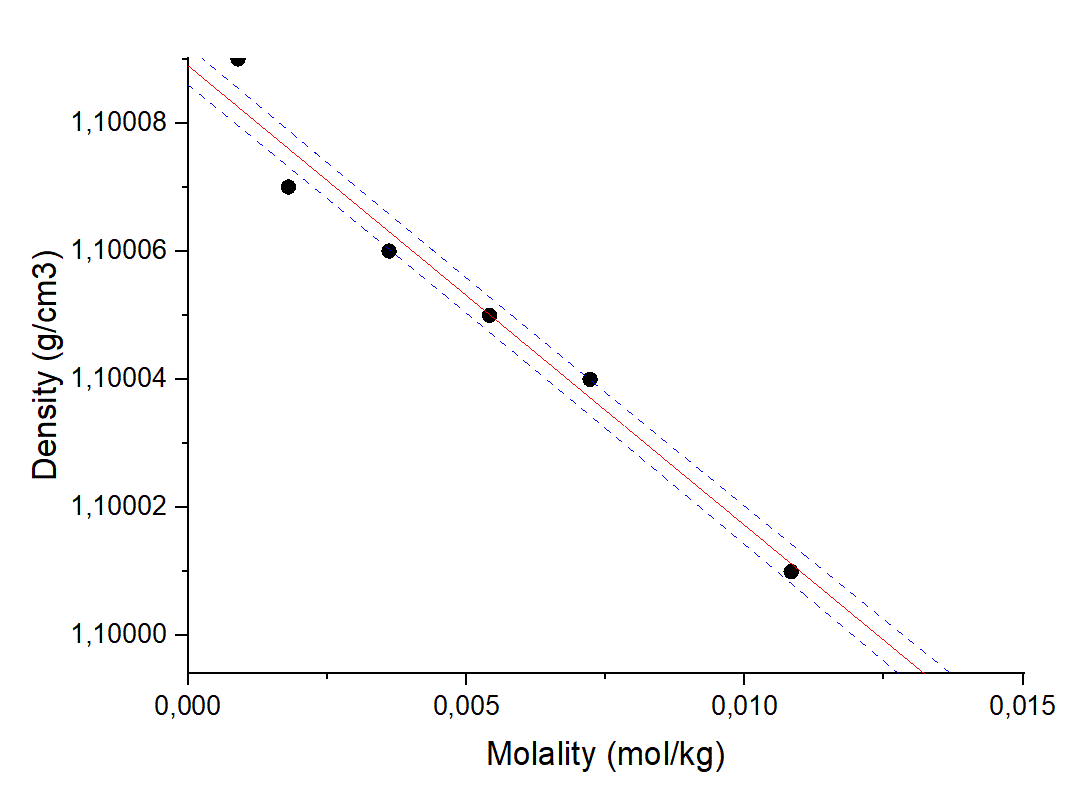

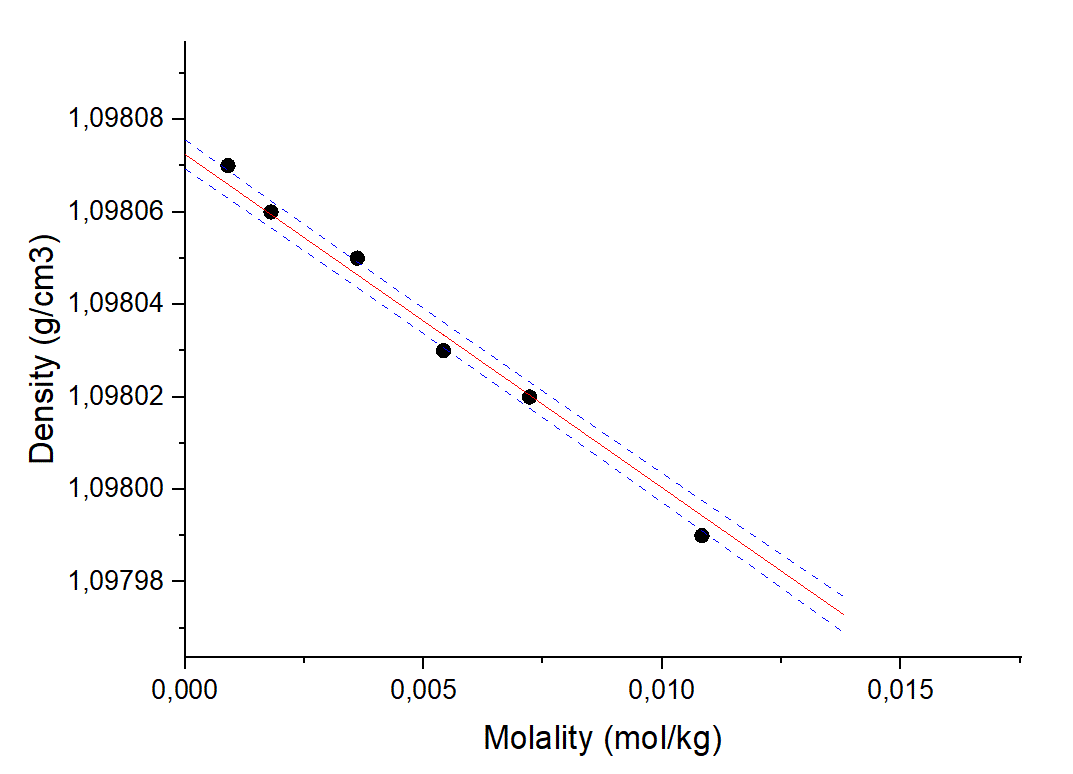


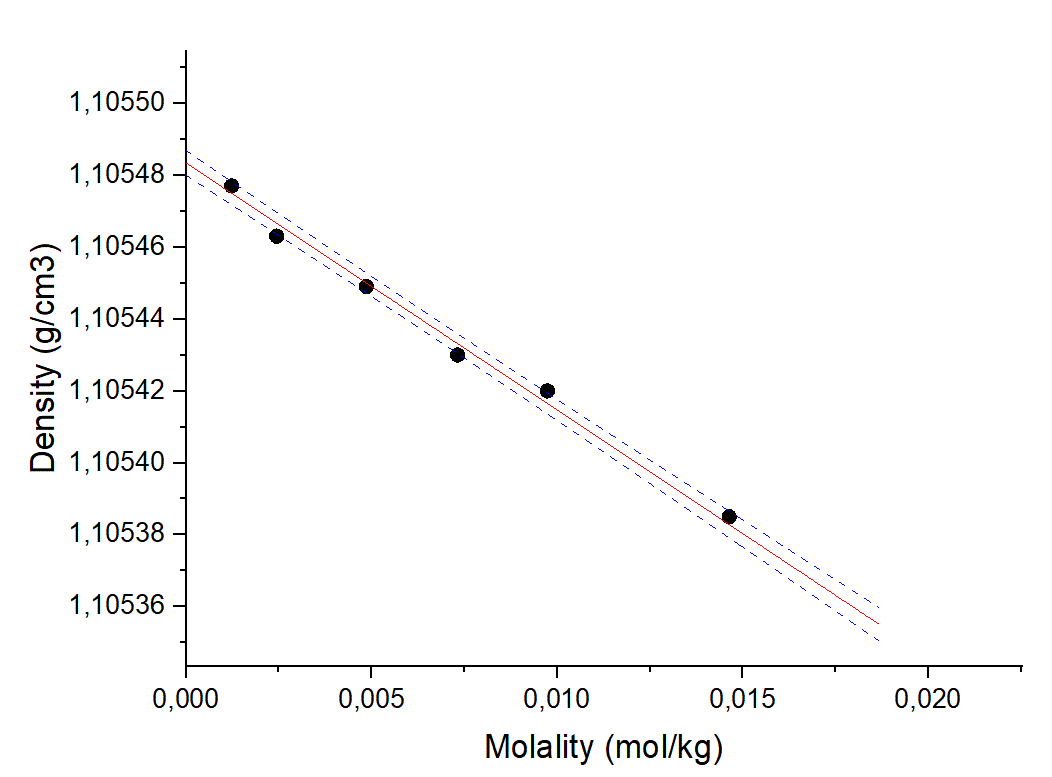

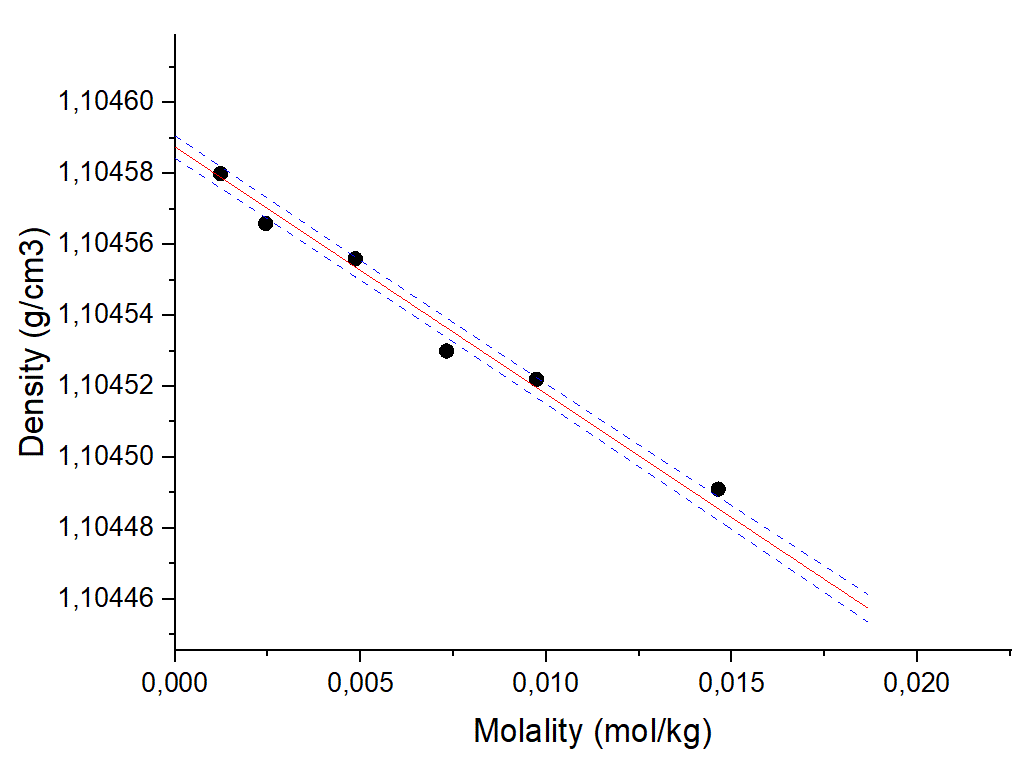

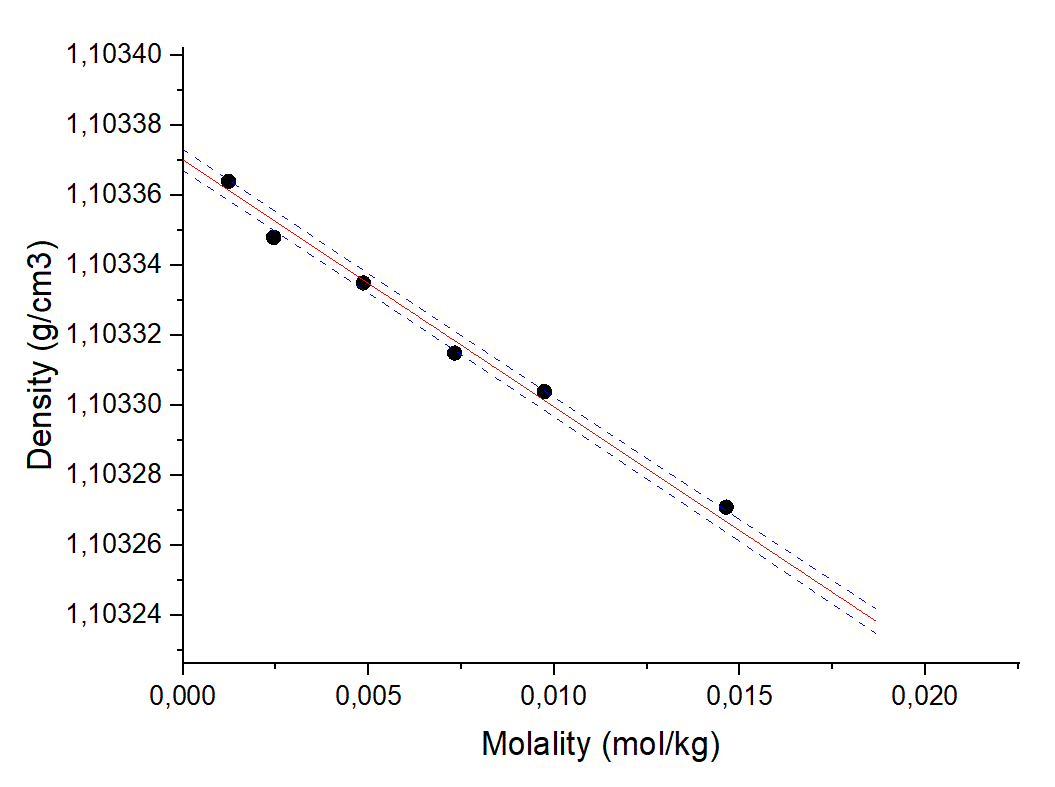


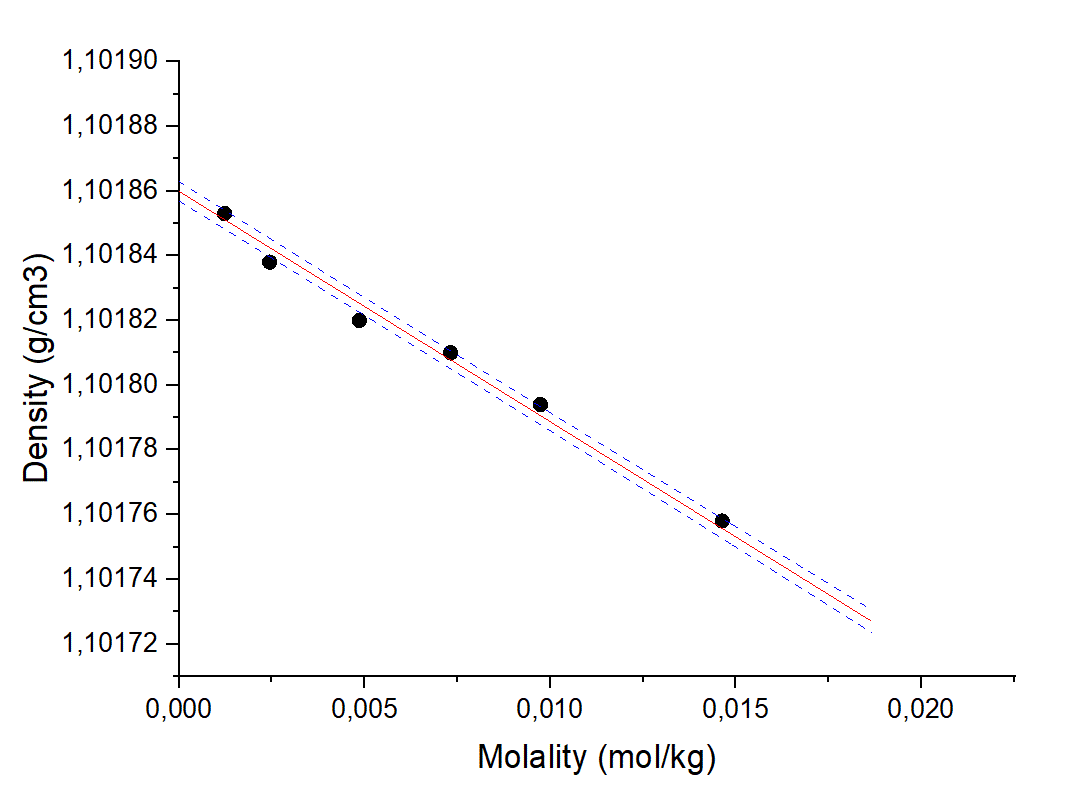

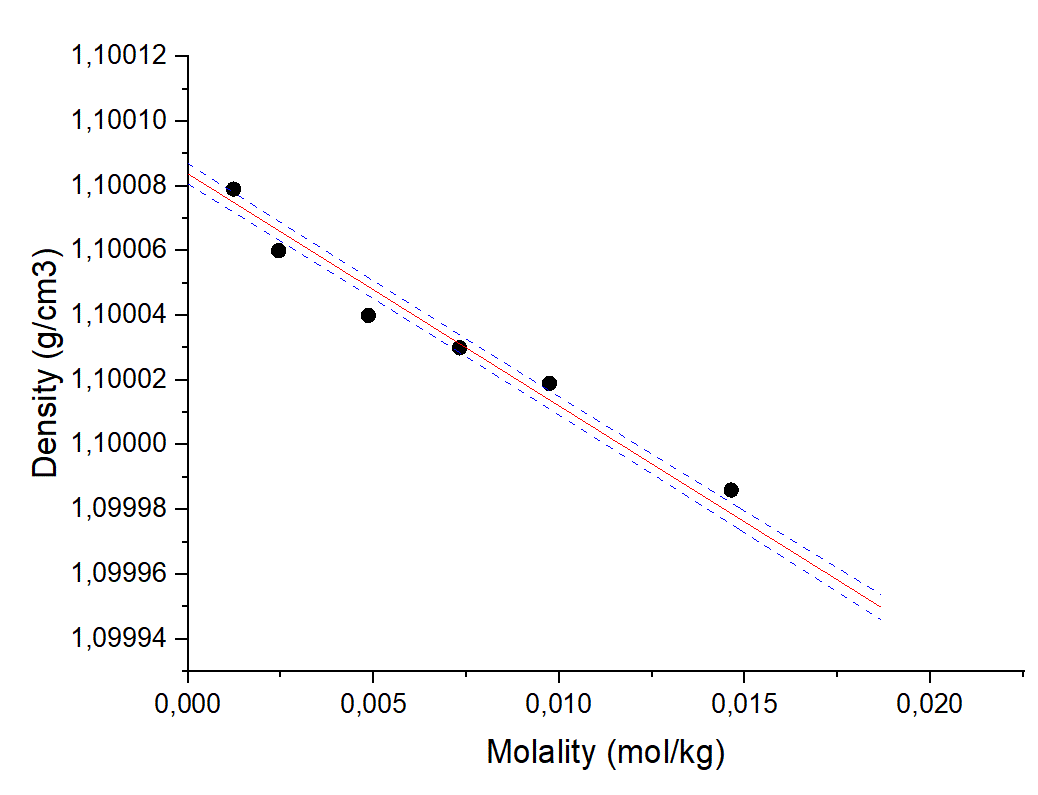

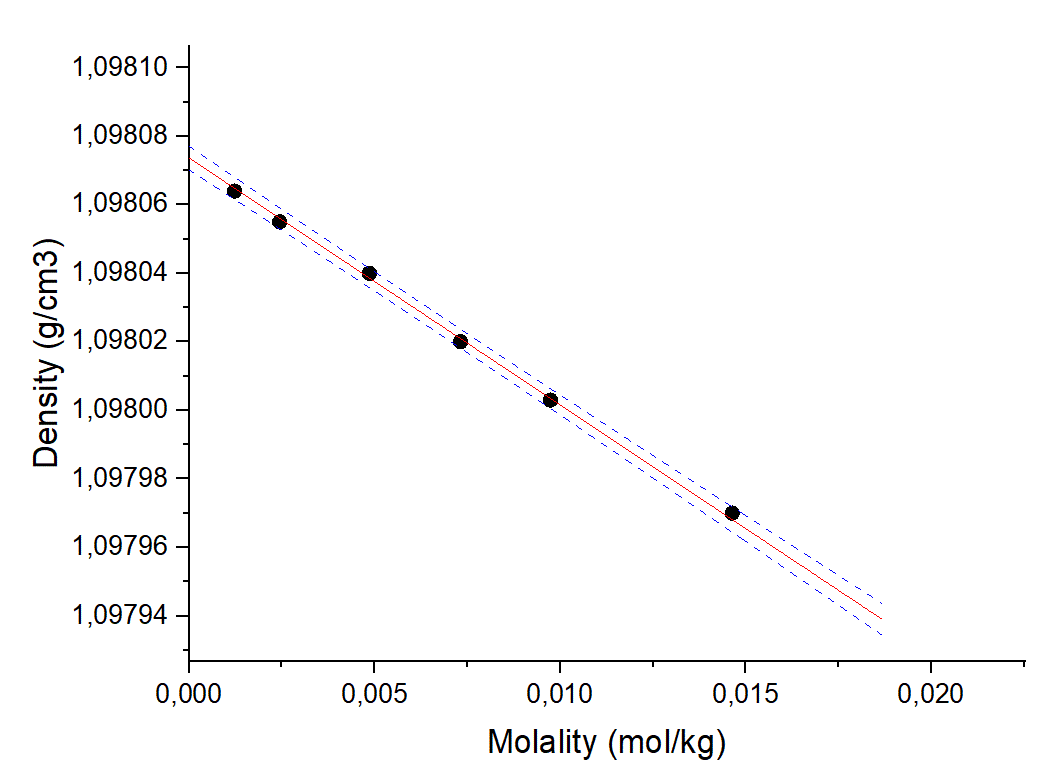


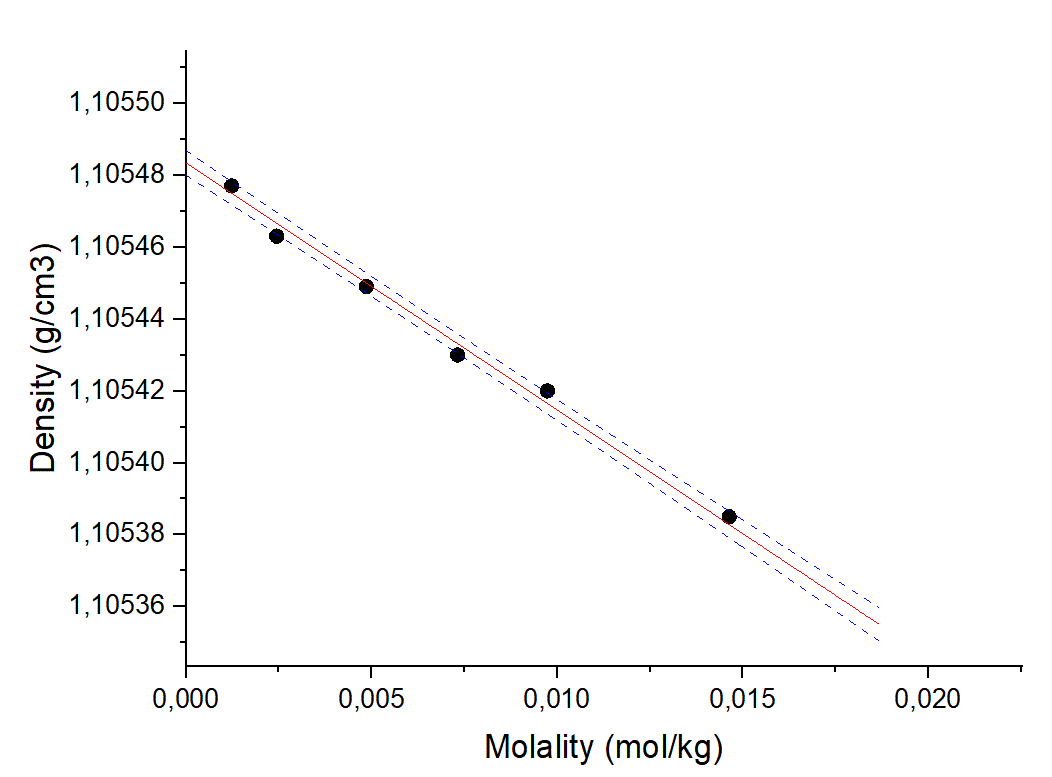

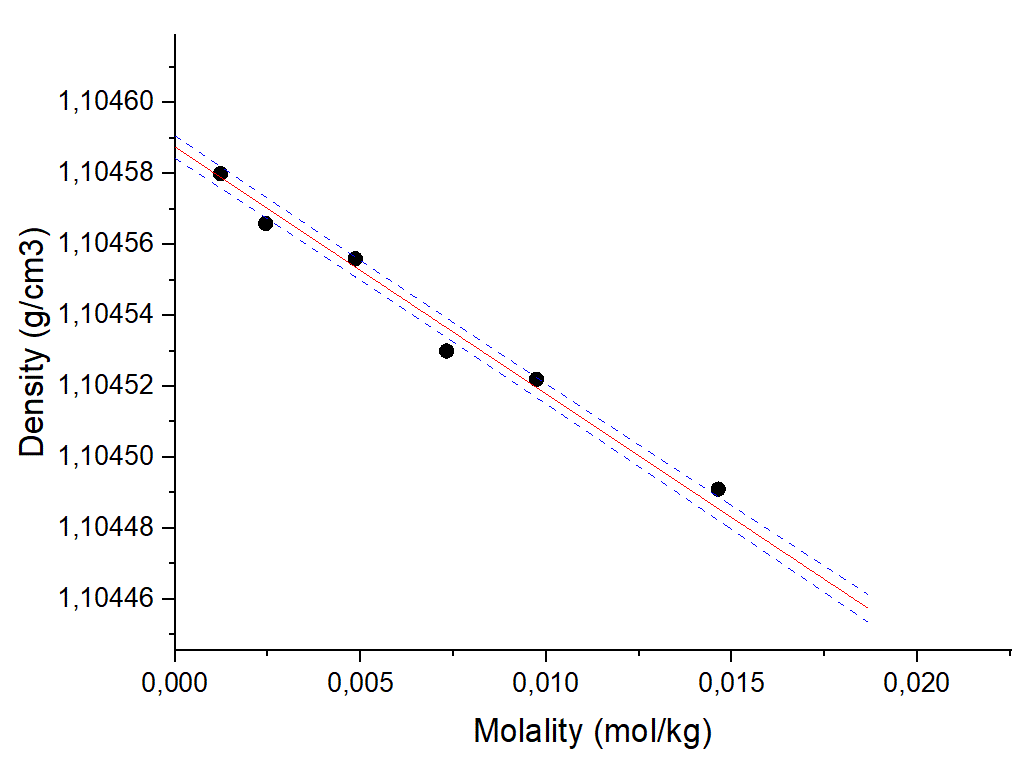

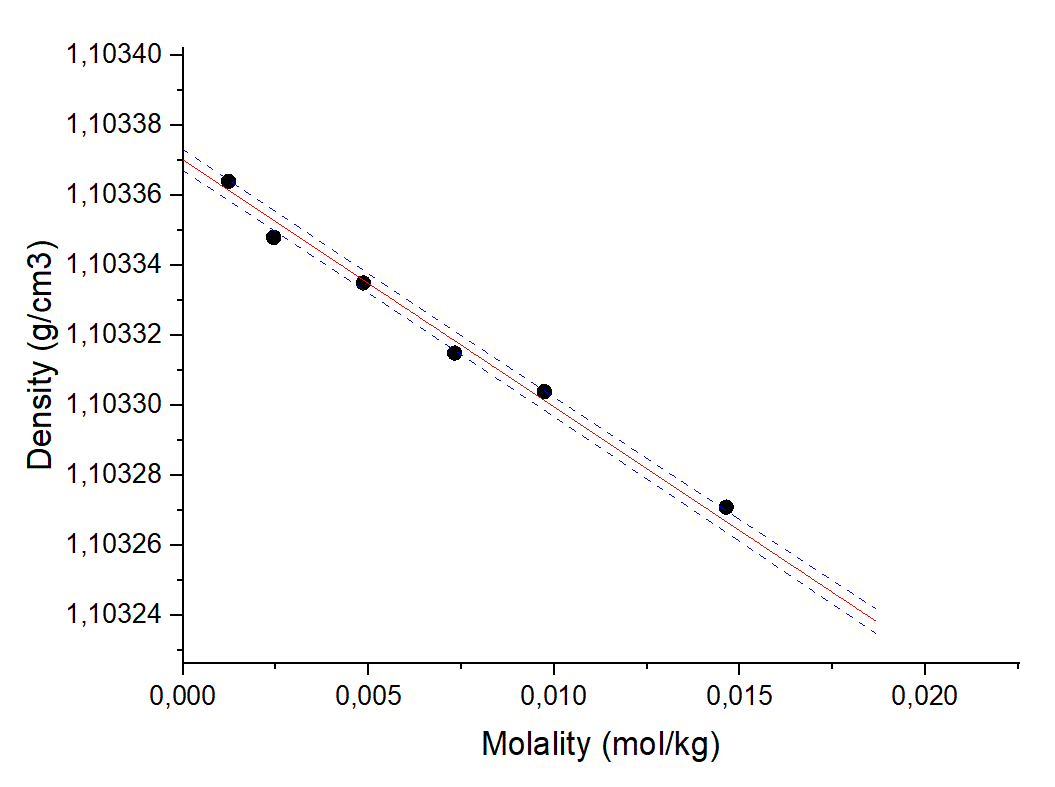


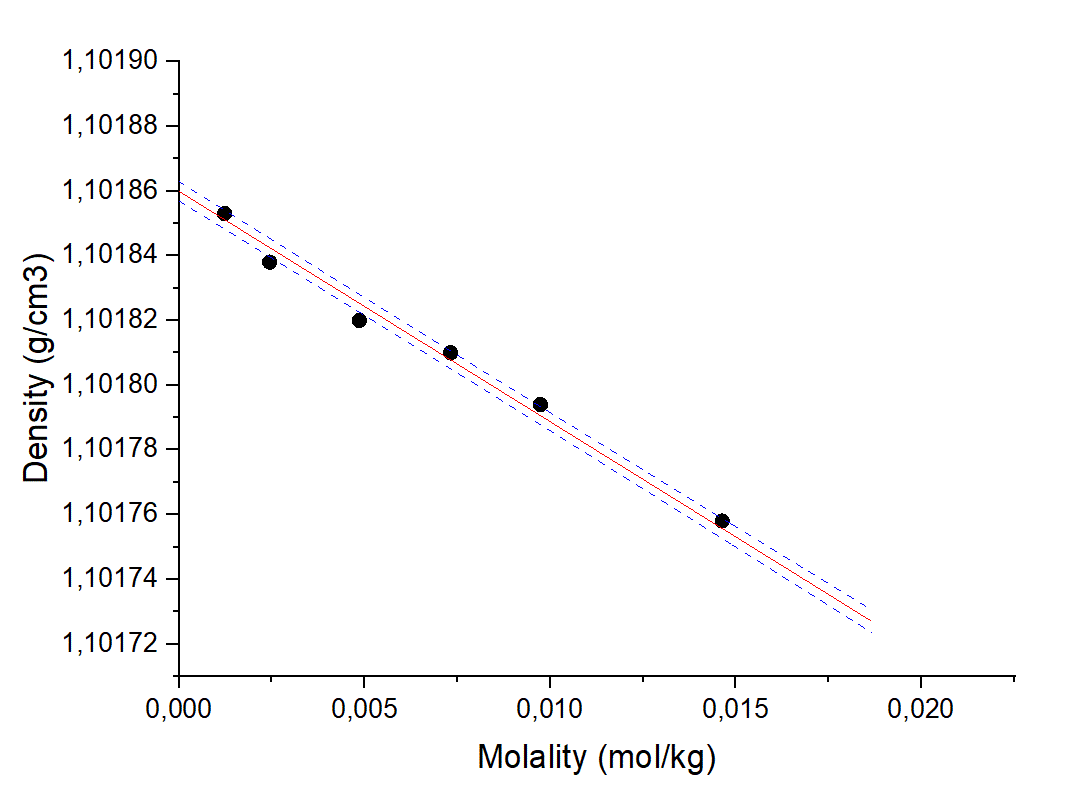

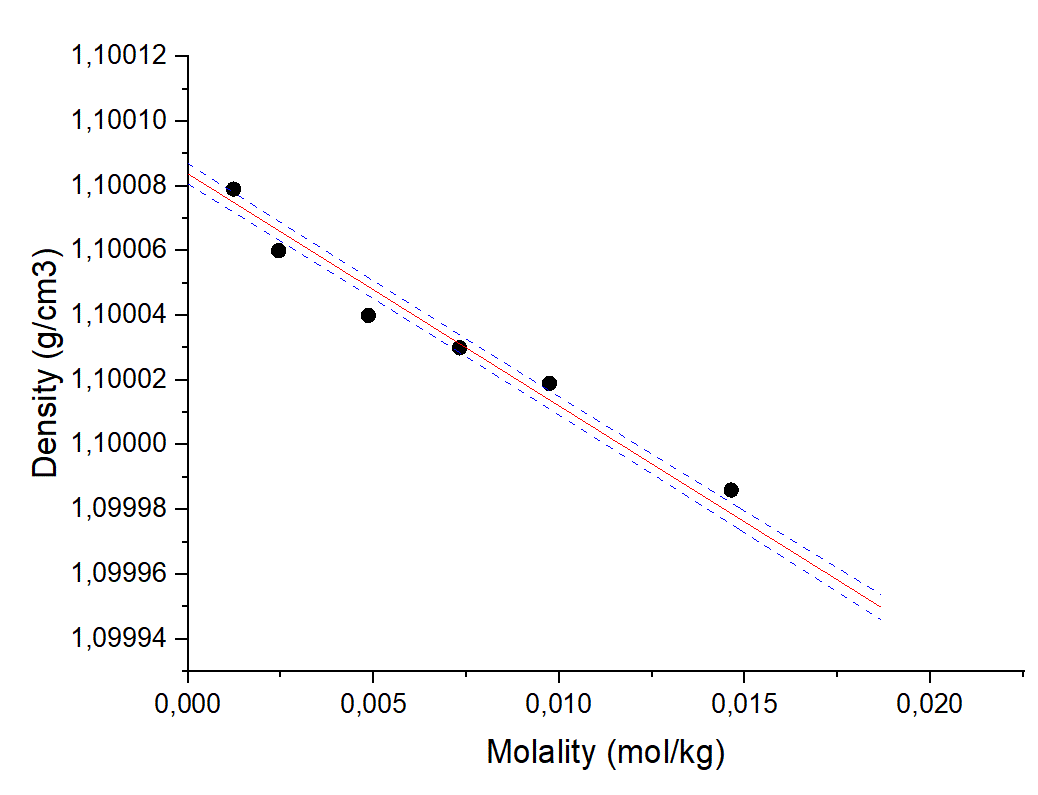

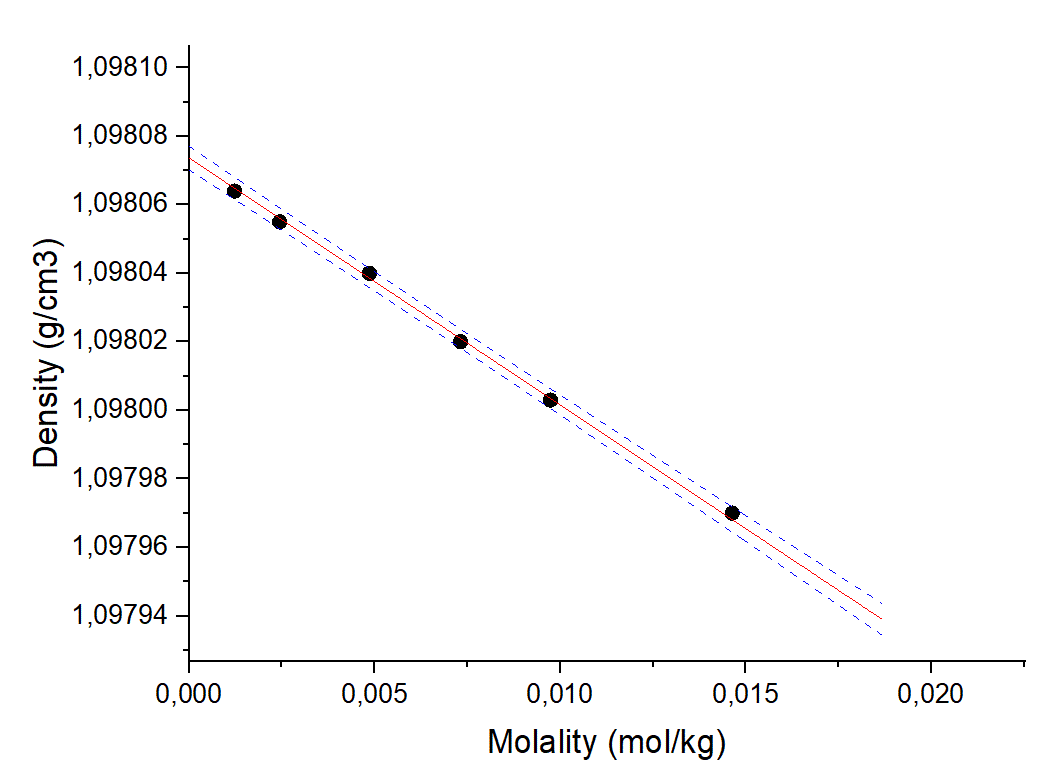


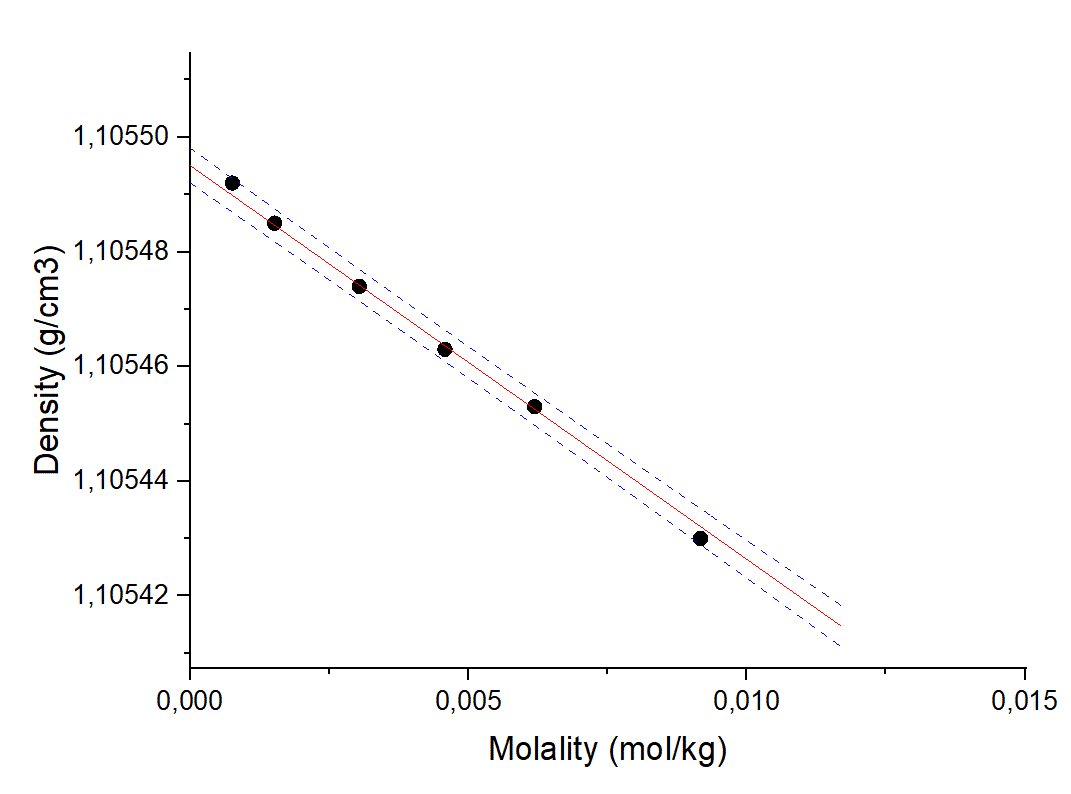

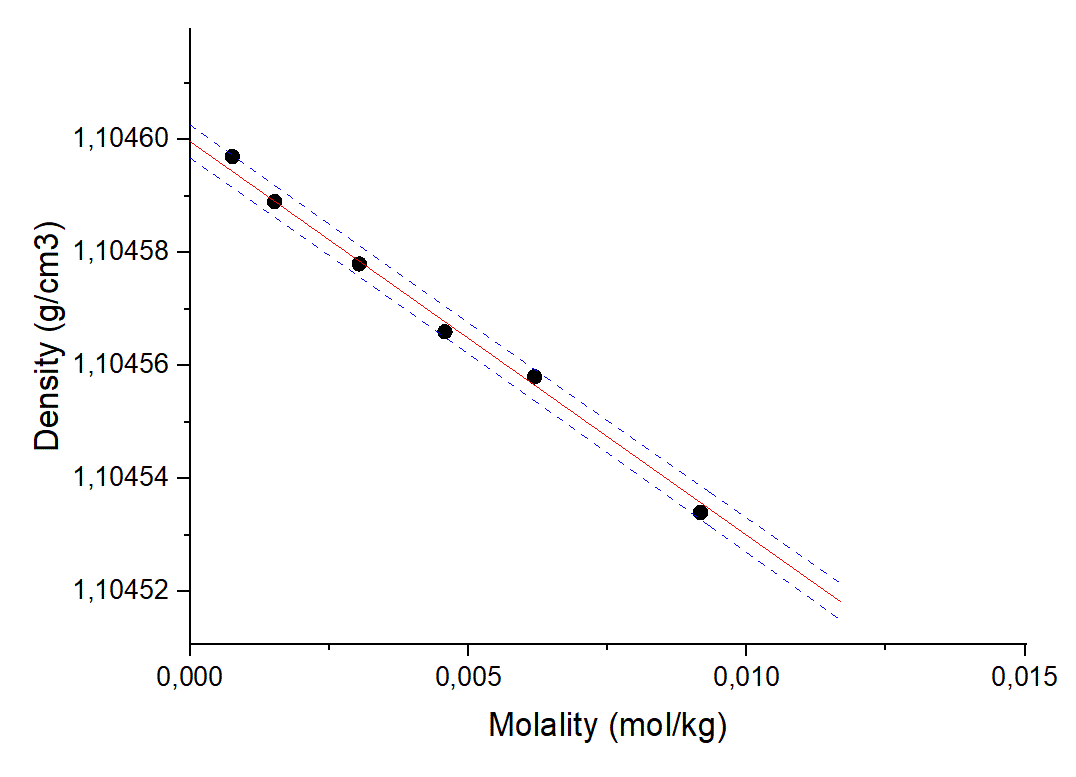

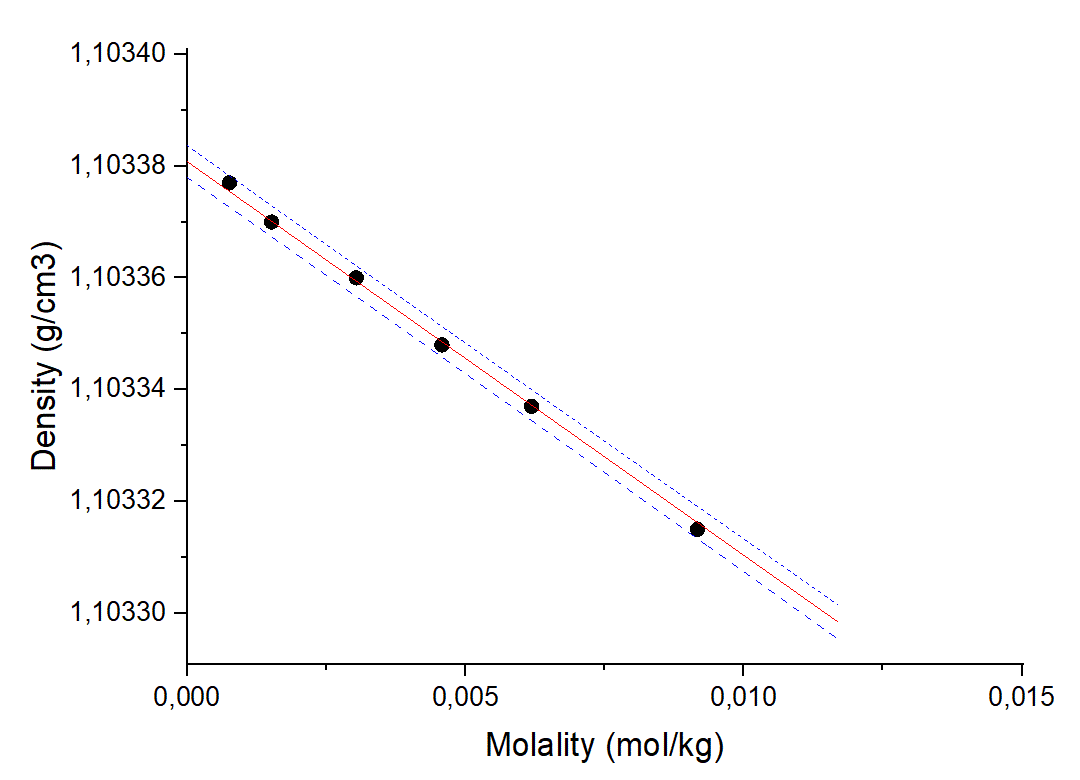


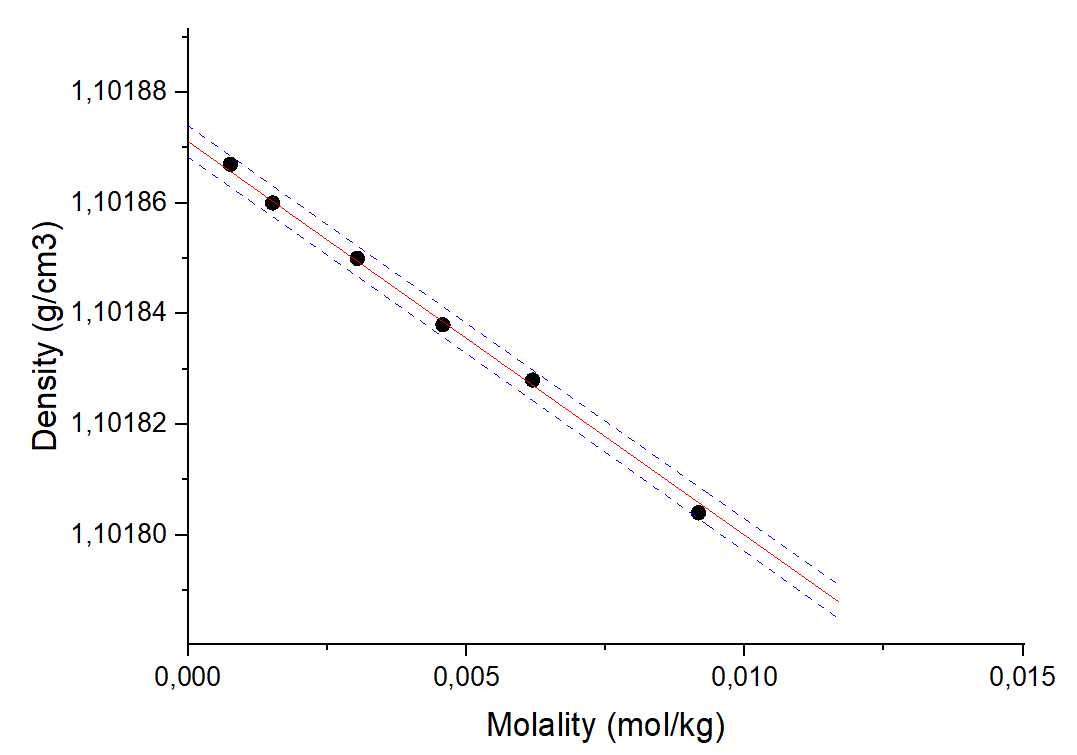

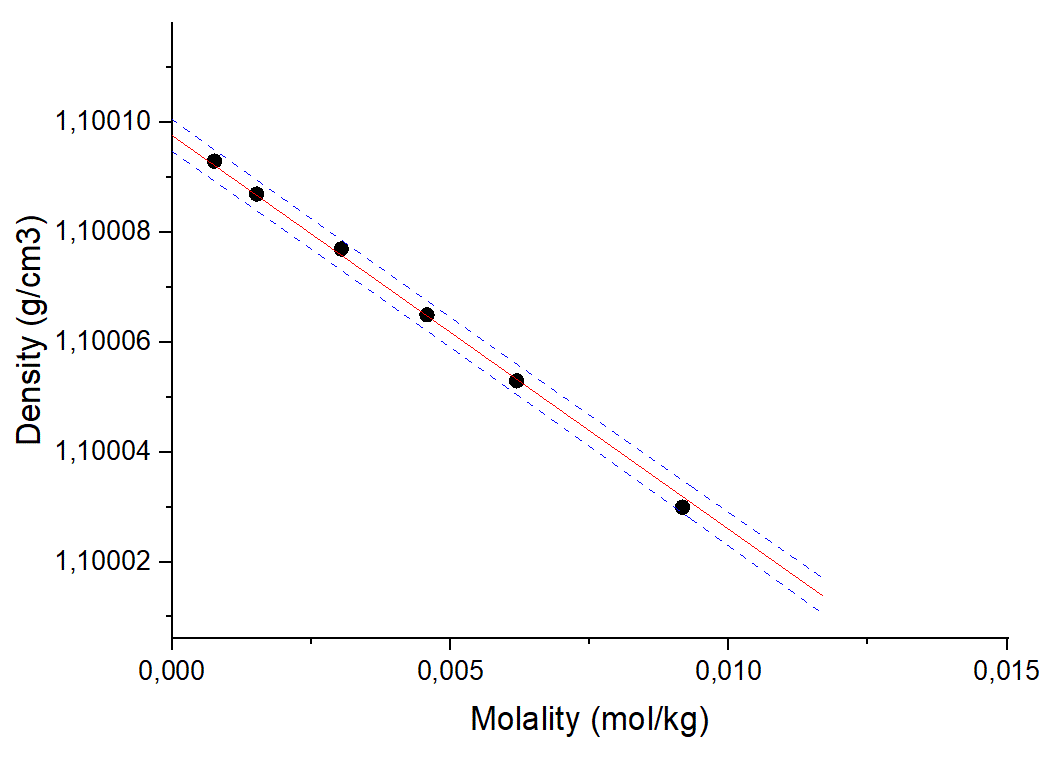

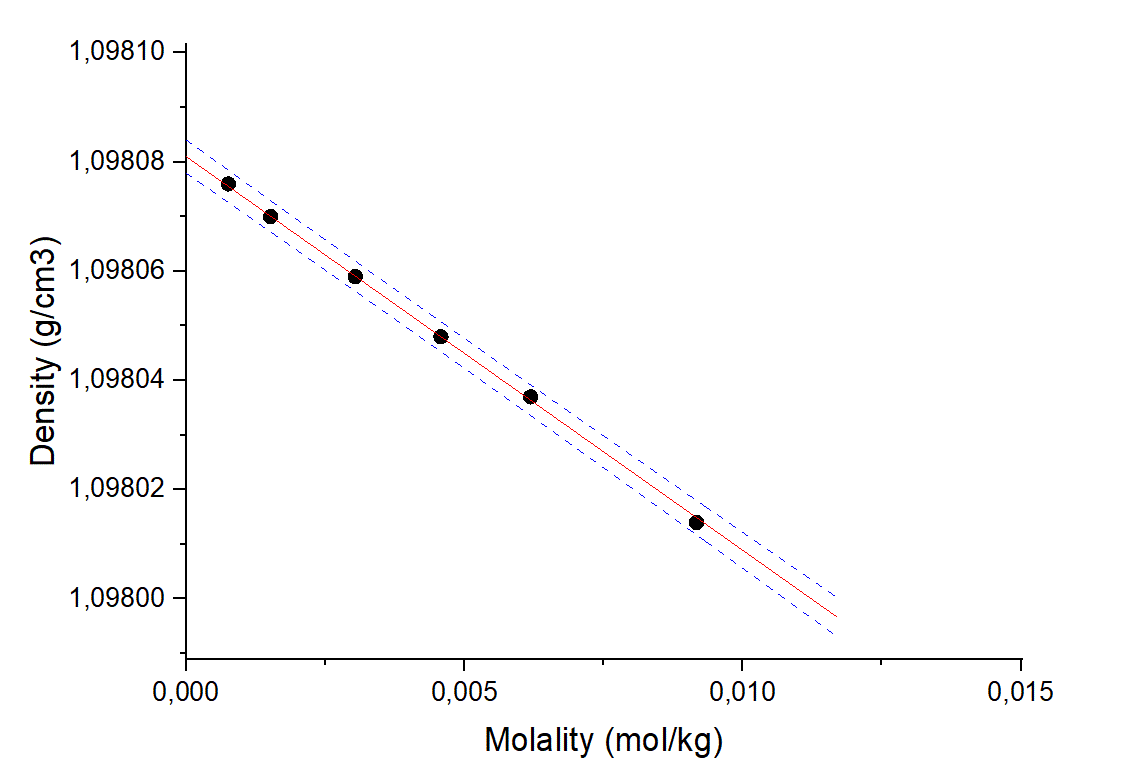


**Figure S2.** Density - molality relationship for HDO in D_2_O measured experimentally for 6 temperatures in range of 20 to 45^o^C. The results of four independent experiments are shown.


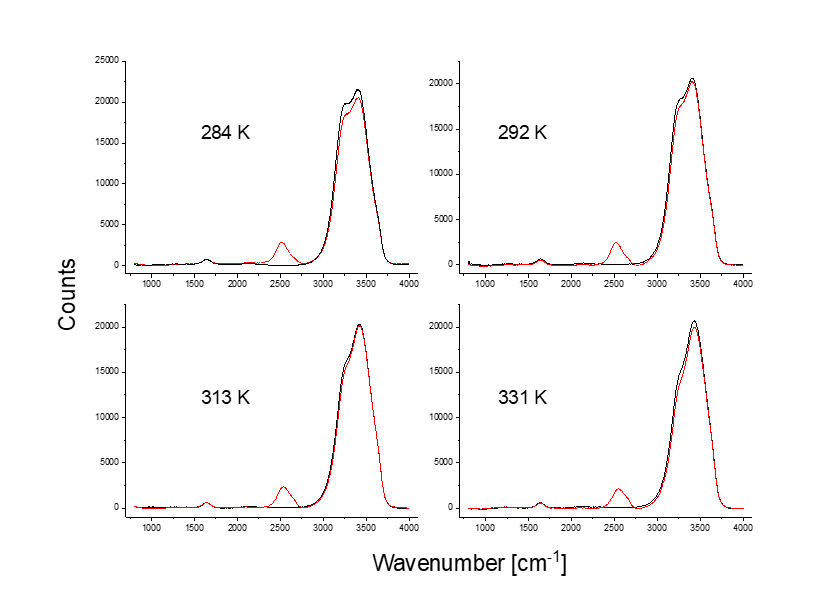


**Figure S3.** Raman spectra of bulk H_2_O (black) and the mixture of 5% D_2_O in H_2_O (red), measured at four different temperatures.

**Figure S4:** Raman spectra of bulk D_2_O (black) and the mixture of 5% H_2_O in D_2_O (red), measured at four different temperatures.

**Figure S5.** Normalized IR absorption spectra of ultrathin (~1,4 - 2 µm) films containing pure H_2_O and mixtures containing 2.5, 5, 7.5, and 10% of D_2_O. The arrow indicates the increasing concentration of D_2_O.


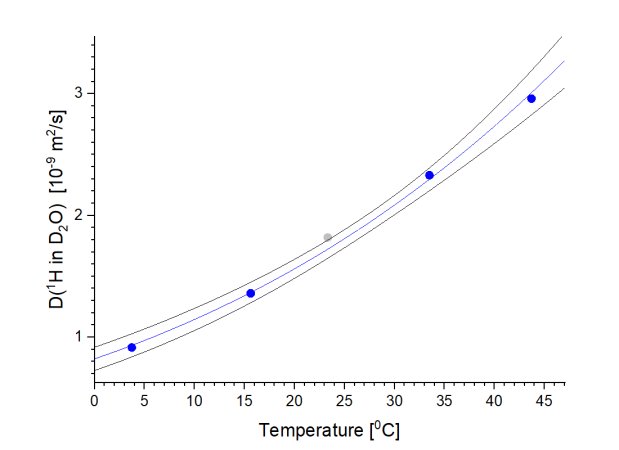

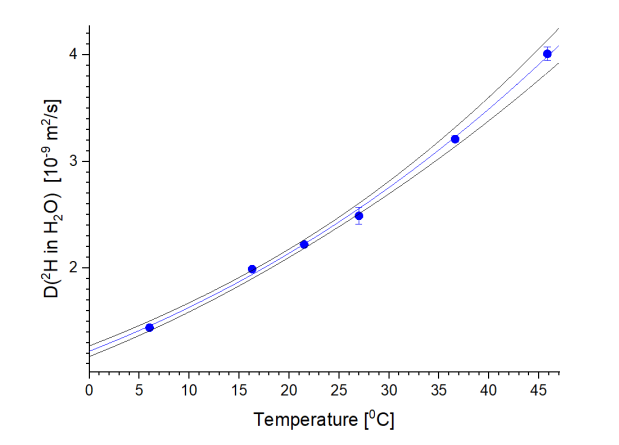


1. **b)**

**c) d)**


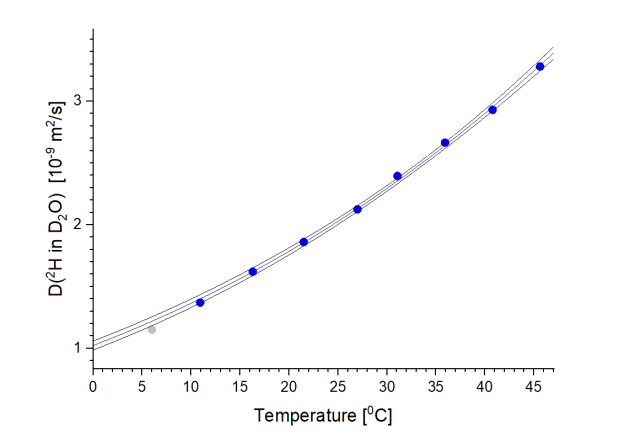

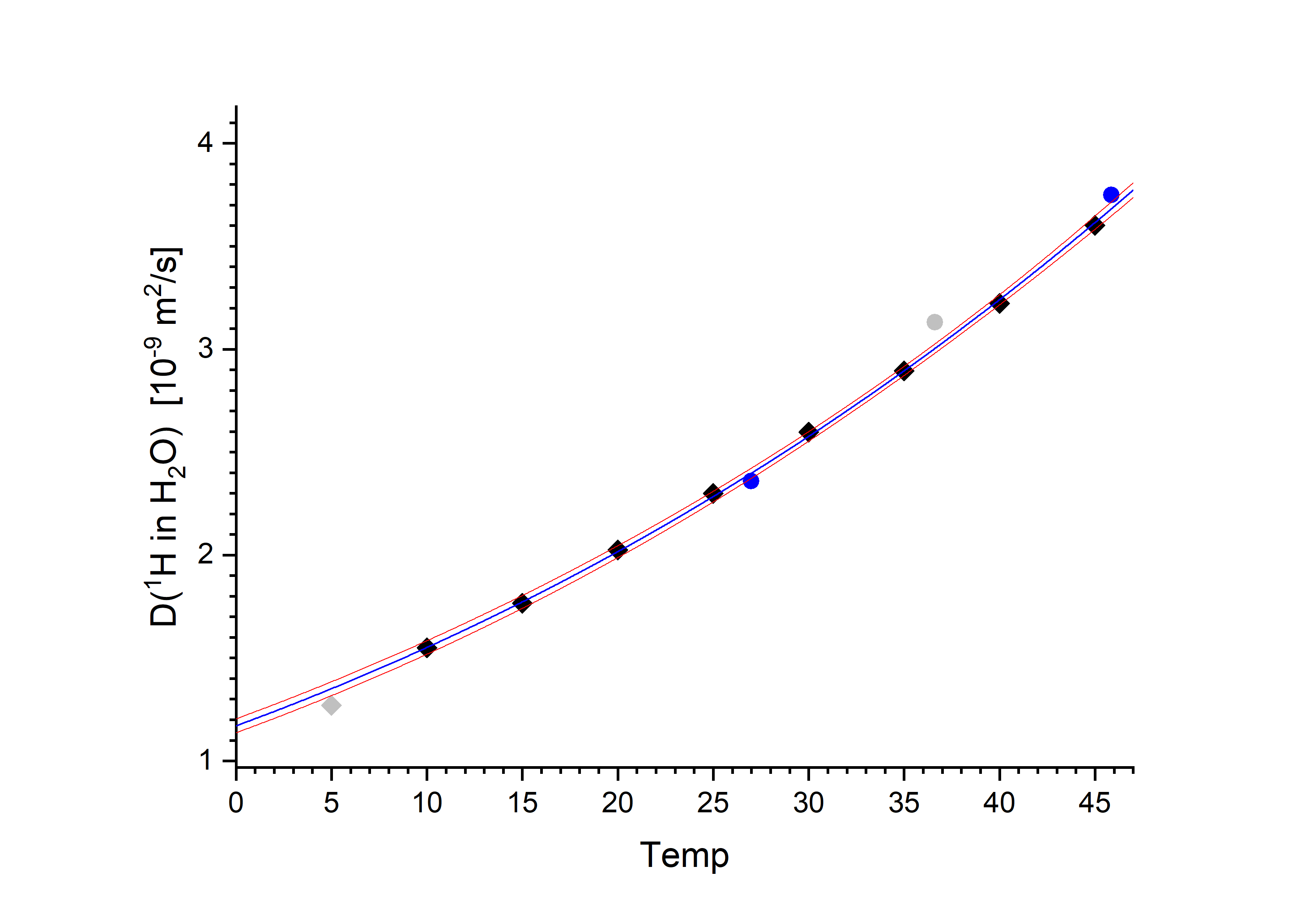


**Figure S6.** Temperature dependence of self-diffusion coefficients for: a) ^1^H in D_2_O, b) ^2^H in H_2_O, c) ^2^H in D_2_O, d) ^1^H in H_2_O. Diamonds represent data taken from ref. 64; points denoted in gray were omitted in the analysis; red lines boarder 95% confidence bands for the fitted model shown in blue.
